# Spatially and Temporally Distinct Encoding of Muscle and Kinematic Information in Rostral and Caudal Primary Motor Cortex

**DOI:** 10.1093/texcom/tgaa009

**Published:** 2020-04-04

**Authors:** James Kolasinski, Diana C Dima, David M A Mehler, Alice Stephenson, Sara Valadan, Slawomir Kusmia, Holly E Rossiter

**Affiliations:** Cardiff University Brain Research Imaging Centre, School of Psychology, Cardiff University, Cardiff, CF24 4HQ, UK

**Keywords:** dexterity, kinematics, motor cortex, movement, MVPA

## Abstract

The organizing principle of human motor cortex does not follow an anatomical body map, but rather a distributed representational structure in which motor primitives are combined to produce motor outputs. Electrophysiological recordings in primates and human imaging data suggest that M1 encodes kinematic features of movements, such as joint position and velocity. However, M1 exhibits well-documented sensory responses to cutaneous and proprioceptive stimuli, raising questions regarding the origins of kinematic motor representations: are they relevant in top-down motor control, or are they an epiphenomenon of bottom-up sensory feedback during movement? Here we provide evidence for spatially and temporally distinct encoding of kinematic and muscle information in human M1 during the production of a wide variety of naturalistic hand movements. Using a powerful combination of high-field functional magnetic resonance imaging and magnetoencephalography, a spatial and temporal multivariate representational similarity analysis revealed encoding of kinematic information in more caudal regions of M1, over 200 ms before movement onset. In contrast, patterns of muscle activity were encoded in more rostral motor regions much later after movements began. We provide compelling evidence that top-down control of dexterous movement engages kinematic representations in caudal regions of M1 prior to movement production.

## Introduction

Mounting evidence supports the encoding of movements in M1 based on kinematics and synergistic muscle activation, rather than the anatomy of the peripheral musculature (Overduin et al. 2012, 2015). Measurements from individual M1 neurons in non-human primates reveal the encoding of multiple kinematic features, such as speed, direction, and position in the same cells in a time-varying manner (Fu et al. 1995). The same neuronal populations have been shown to encode instantaneous features during motor execution, as well as the target kinematic end point and upcoming movement trajectory (Churchland and Shenoy 2007; Hatsopoulos et al. 2007; Aflalo and Graziano 2006; Saleh et al. 2012).

In the human brain, evidence of neuronal tuning to multiple kinematic features has been reported during the production of intended movements from M1 microelectrode recordings made in tetraplegic patients (Truccolo et al. 2008). The encoding of kinematic features of hand movements in M1 has also been supported by human imaging studies (Dayan et al. 2007; Kadmon Harpaz et al. 2014, 2019). Patterns of functional magnetic resonance imaging (fMRI) activity in sensorimotor cortex have been shown to mirror the relative differences in the final joint configuration across a range of prehensile movements (Leo et al. 2016). Similarly, the representational structure of fMRI activity in M1 during finger flexion is consistent with patterns of finger couse during naturalistic hand movements (Ejaz et al. 2015).

However, the functional relevance of kinematic encoding in M1 to human motor control remains a fundamental unknown. As well as their role in motor output, M1 neurons exhibit rapid and integrative responses to somatosensory signals (Hatsopoulos and Suminski 2011; Pruszynski et al. 2011). Kinematic information is inextricably linked to proprioceptive and tactile signals: specific patterns of movement are associated with specific patterns of sensory feedback. Are kinematic motor representations reported in human M1 functionally relevant in the process of top-down motor control, or an epiphenomenon generated by bottom-up sensory feedback during human movement production?

We addressed this question using a spatiotemporal multivariate representational similarity analysis (RSA) to ask where in the human brain and when during movement production are the kinematics of human hand movements encoded? This approach combined high-field fMRI and magnetoencephalography (MEG) data with kinematic data glove recordings made during a broad repertoire of prehensile and nonprehensile hand movements. Probing recordings of human brain activity with high spatial resolution from fMRI and high temporal resolution from MEG offered a powerful means to identify the location and timing of kinematic information encoding. Together this information was used to dissociate the relevance of kinematic information in M1 to top-down or bottom-up processes in motor control, as well as the relevance of alternative muscle-based or ethological action based models.

## Materials and Methods

### Methods Summary

A total of 10 right-handed participants performed a range of 26 prehensile and nonprehensile hand movements (Elliott and Connolly 1984; Jones and Lederman 2006) (Table 1, Supplementary Video S1) in two fMRI sessions (1.5 h total fMRI data per participant), two MEG sessions (1.5 h total MEG data per participant), and a behavioral testing session (35 min kinematic data recording per participant). In each session, participants wore a right-handed 14-channel fiber optic data glove; kinematic data were recorded throughout all sessions. Electromyography (EMG) data were acquired during MEG sessions to validate the movement onset measures calculated from the data glove.

**Table 1:** Outline of the 26 hand movements used in the motor task. Instructional videos presented in Supplementary Video S1

Hand movements	
Abduct fingers	Pinch: thumb and little finger
Cylinder grip	Pinch: thumb and index finger
Hook grip	Pinch: thumb and middle finger
Spherical grip	Pinch: thumb and ring finger
Index finger flexion (45°)	Ring finger flexion (45°)
Index finger flexion (90°)	Ring finger flexion (90°)
Index & middle finger flexion (90°)	Ring and little finger flexion (90°)
Index finger and thumb roll	Rock fingers
Little finger flexion (45°)	Squeeze: thumb and fingers
Little finger flexion (90°)	Abduct thumb
Middle finger flexion (45°)	Extend thumb
Middle finger flexion (90°)	Flex thumb
Middle & ring finger flexion (90°)	Twiddle: thumb and index finger

**Figure 1:**
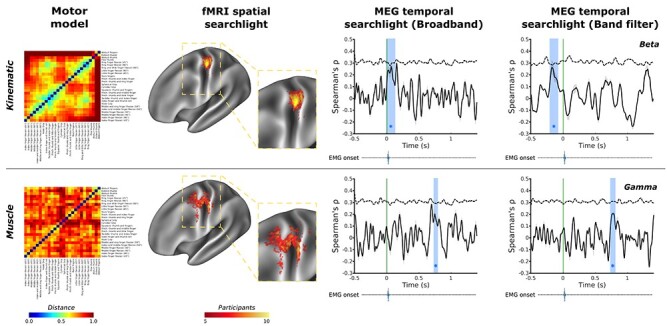
Spatial and temporal evidence for distinct encoding of kinematic- and muscle-based information in human motor cortex. Group average kinematic and muscle models of hand movement were used in a spatiotemporal RSA. Top row: fMRI data show that kinematic information was encoded consistently in of primary motor cortex across all 10 participants with a consistent peak in Brodmann areas 4 and 3a; complementary MEG data revealed temporal encoding of kinematic information (blue box) around the point of movement onset in the broadband signal, further decomposition of which revealed encoding prior to movement onset (green line) in the beta frequency, from −210 to −90 ms. The group average muscle model (bottom row) showed consistent spatial encoding in more rostral regions of Brodmann area 4 of primary motor cortex across participants, as well as postcentral regions of Brodmann areas 3b; a temporal searchlight using the muscle model revealed evidence of encoding much later in the cycle of movement around 735–795 ms after movement onset in the broadband signal; further decomposition revealed this encoding of the muscle model originated in the gamma frequency. An ethological action model in line with recent primate studies (Graziano, 2016) was investigated and is presented in Supplementary Figure S18. Full MEG analysis are presented in Fig. 3. Green line—movement onset defined by the data glove; blue regions—significant peaks in representational similarity between MEG data and the motor model; dashed line—correlation noise ceiling. EMG onset violin plots based on data presented in Supplementary Figure S10. Both matrices are presented based on the results of hierarchical clustering conducted on the kinematic matrix for ease of comparison. Both model matrices are reproduced in a larger format in Supplementary Figure S2 using their own respective hierarchical clustering outputs.

To probe the spatial and temporal correspondence between patterns of brain activity and hand kinematics, data glove recordings were used to construct a kinematic model quantifying the similarity of the kinematic signals measured during each of the 26 movements (Fig. 1, top row, Supplementary Figure S2). The kinematic model quantified the distance between the displacement measures for each movement pair across the 14 channels (Pearson’s correlation), subject to a Fisher Z-transformation and averaged across the 14 recording channels. The resulting kinematic model exhibits strong split-half and intersession consistency within participant (Supplementary Figure S1). In both the spatial and temporal RSA, the kinematic model was investigated alongside two other models. A muscle-based model was constructed from high-density EMG recordings (15 channels) made in an independent cohort of 10 participants performing the same range of hand movements (Fig. 1, bottom row). An additional ethological action model classified movements into precision prehensile, power prehensile, and nonprehensile, based on the notion of ethological maps in primate M1 (Elliott and Connolly 1984; Graziano 2016) (Supplementary Figure S18). A group average kinematic and muscle model were subject to nonclassical multidimensional scaling for visualization of the relative dissimilarity of each movement across three dimensions (3D Graphics 1 and 2). An equivalent analyses in two dimensions using videos illustrating the various movements is also presented for the group average kinematic model (SupplementaryVideo S2).

### Participants and Experimental Design

All data were acquired according to the local university research ethics committee approval in line with the Declaration of Helsinki (Cardiff University School of Psychology Research Ethics Committee: EC.17.03.14.4874 and EC.17.04.11.4885) All participants provided written informed consent and met local MRI and MEG safety criteria.

A total of 10 right-handed participants were recruited in the main study (age range:22–30; mean age: 24.0; Age SD: 2.8; 5 females). Participants were not currently taking any psychoactive medications, and were right-handed according to the Edinburgh Handedness Inventory (Oldfield, 1971). No participants had a history of any disorder affecting tactile sensory or motor function or any history of neurological illness. Each participant undertook five experimental sessions: two MRI scan sessions, two MEG recording sessions, and one behavioral testing session. All participants undertook the behavioral testing session first; the subsequent order of the fMRI and MEG sessions was counterbalanced, leaving a minimum of 2 weeks between any one MRI and MEG session to minimize the effects of magnetic noise on the MEG signal (Gross et al. 2013). The datasets generated and analyzed during the current study are available from the corresponding author on reasonable request.

### Motor Task and Kinematic Data Acquisition

During all sessions participants were engaged in a motor task involving the production of a range of 26 hand movements (Table 1, Supplementary Video S1) with the right hand while wearing a fiber-optic kinematic data glove (Data Glove 14 Ultra; Fifth Dimension Technologies: 5DT, Orlando, FL, USA). Kinematic data were acquired across 14 independent fiber-optic channels (one proximal and one distal sensor per digit, plus one sensor between each digit pair) at 60 Hz. Flexion, extension, pitch, and roll cause deformation in the fiber optic channels, impacting the transmission of fiber optic signals and generating a quantifiable signal change. The behavioral task using the data glove was implemented in PsychoPy (Version 1.84.20) (Peirce, 2007, 2009) using the Python Computer Graphics Kit (CGkit: cgkit.sourceforge.net) SDK wrapper for the 5DT data glove.

Each recording session was divided into task runs; each task run was composed of blocks of a specific movement; each block comprised individual movement trials; details of the number of runs, blocks, and trials are specified for MEG and fMRI sessions, respectively, below. Instructions were presented on a screen in the testing environment. Each task run contained one block of each of the 26 movement types, ordered using a random-without-replacement selection method. Progressive determination effects were minimized by maximizing the range of different conditions in each run; presenting all 26 movements once per run (Blais, 2008). At the beginning of each movement block, participants were shown a 3 s video of the movement to be produced (Supplementary Video S1). Participants were cued to produce the movement in question in each subsequent movement trial of the block by an expanding and contracting horizontal bar. In each movement trial, the bar began at a fully contracted width, colored red, indicating that the hand should be static and in a resting flat position. The bar subsequently turned green and began to expand symmetrically at its left and right flanks. Once it reached its maximal width, the bar began to contract back to its original width. Once the bar reached its original contracted width, it turned red, signifying the end of the movement trial. Participants were instructed to pace their movements to coincide with the period of expansion and contraction of the green bar, such that their hand assumed a flat position at the beginning and end of each trial, corresponding to the time that the static red bar was presented. The motor task was conducted in a behavioral testing lab, in the MRI scanner, and in the MEG scanner, as detailed below.

None of the grasping tasks in this study engaged participants with real objects; previous work has differentiated motor activity with or without real objects in anterior intraparietal sulcus, but not primary motor cortex: as such an object-free study design seemed appropriate for a study focusing on M1 (Freud et al. 2018).

### Kinematic Recording Session

During the behavioral testing session participants performed five runs of the motor task. Participants were seated at a desk with their right forearm supported on a memory foam mount, while wearing the data glove. Participants viewed instructions presented on a 14 inch laptop display. Each movement block comprised a 3 s video of the movement to be produced, a 1 s preparation period and 8 subsequent movement trials; each comprising 1.6 s of movement (green expanding/contracting bar), followed by a 0.8 s rest period (red static bar). The transition of the bar from red to green was defined as the go signal. A break period of up to 15 s was permitted between each movement block; participants advanced the task with a keypress using their left hand. Excluding break periods each task run was 10 min and 3.2 s in duration. The five task runs yielded 33 min and 16.8 s of kinematic data recording per participant.

### Kinematic Movement Model

For each participant, kinematic data from the behavioral, MRI, and MEG sessions were each processed in parallel. This yielded a separate kinematic model from each session type for each participant. These models were used in subsequent multivariate fMRI and MEG analysis; they captured the kinematic similarities and differences of the 26 distinct movements under study.

Initially the kinematic data from each session and each movement block were epoched into individual movement trials using the time of onset of the green bars and averaged. The resulting 14 channels of data represented the average pattern of displacement of the hand during a movement trial for a given movement, termed the kinematics of the movement: the motion of the hand without reference to the forces that produce this motion. In order to compare this signature of kinematic activity for each possible pairing of the 26 movements, the activity pattern of each of the 14 recording channels was correlated channel-wise using Pearson’s correlation coefficient, subject to the Fisher *Z*-transformation, and the resulting values were averaged across channels to yield a single measure of the similarity of kinematics across each movement pair. The resulting value was transformed back into a Pearson’s r-value and used to construct a 1-r dissimilarity matrix for each movement pair.

The kinematic dissimilarity matrices were averaged across task runs to yield an average fMRI, MEG, and behavioral kinematic model for the group. The split-half consistency and intersession consistency of these models is outlined in Supplementary Figure S1. A grand average across all sessions and participants was computed and subjected to hierarchical clustering; this resulting clustering was applied to visualizations of the kinematic model and the muscle model (Fig. 1). Clustering for the group average muscle model is presented in Supplementary Figure S2. All analyses used the group average muscle and kinematic models.

### Muscle Model

An independent EMG dataset was acquired in order to construct a model of movement dissimilarity on the basis of muscle activity in the hand. An independent cohort of 10 participants (age range: 20–30; mean age: 25.1; age SD: 3.57; 5 female) undertook a more detailed EMG recording than was feasible during the MEG session, while performing the same 26 hand movements. EMG data were acquired using a Biosemi Active 2 system with a 32 channel headbox (Biosemi B.V. Amsterdam). Muscle activity was recorded using touchproof flat active electrodes. Electrodes 1–15 were placed as labelled in Supplementary Figure S16 closely matched to previously published montages (Ejaz et al. 2015; Leo et al. 2016), namely, first dorsal interosseus (FDI), dorsal interosseus muscles, abductor digiti minimi (ADM), abductor pollicis brevis (APB), adductor pollicis, lumbrical muscles, flexor carpi ulnaris, flexor carpi radialis, flexor digitorum superficialis and flexor digitorum profundus, flexor pollicis longus. Electrode 16 was used to rereference the EMG data in subsequent analysis and was placed on the lateral bony protrusion of the elbow. There were also Common mode sense (CMS) and Driven Right Leg (DRL) electrodes, which served as a ground/reference during recording in the Biosemi software; they were placed on the dorsal aspect of the wrist. The EMG data were recorded at 2048 Hz.

The EMG recording sessions mirrored the design and setup of the kinematic recording session outlined above and were informed by previous fMRI kinematics studies (Ejaz et al. 2015; Leo et al. 2016). Five runs were recorded in total, each containing 26 trials (one for each of the movements). The EMG data were processed using Fieldtrip (Oostenveld et al. 2011). EMG data were rereferenced to electrode 16, rectified and subjected to a band-pass filter (20 Hz and 1000 Hz); and epoched relative to earliest measured muscle onset in any EMG channel using an adaptive threshold (activity duration threshold: 200 ms; 5 ms window smoothing was applied) (Hooman Sedghamiz: Matlab File Exchange: Automatic Activity Detection in Noisy Signals using Hilbert Transform). This resulted in individual trials of 2.0 s in duration. These trials were baselined using the fixation cross window at the start of each trial. EMG trial data were then subject to multivariate noise normalization by weighting channels in trial by the error covariance across the different channels in order to more accurately quantify the true differences between the muscle activity across different movements (Walther et al. 2016; Guggenmos et al. 2018). As in the construction of the kinematic model, the activity pattern of each of the EMG recording channels was correlated channel-wise using Pearson’s correlation coefficient, subject to the Fisher *Z—*transformation, and the resulting values were averaged across channels to yield a single measure of the similarity of kinematics across each movement pair. The resulting value was transformed back into a Pearson’s r-value and used to construct a 1-r dissimilarity matrix for each movement pair. A group average muscle model calculated across all 10 participants’ data was generated and used to probe the spatial and temporal encoding of muscle based dissimilarities in the brain using fMRI and MEG (Fig. 1 and Supplementary Figure S2).

### Ethological Action Movement Model

An alternative ethological action based model was constructed on more recent evidence of ethological maps in primate M1 (Graziano 2016), and therefore categorized movements on the basis of their specific action, namely prehensile movements, subcategorized into precision grip, power grip, and nonprehensile movements (Jones and Lederman 2006) (Supplementary Figure S18). The ethological action model was subjected to hierarchical clustering for visualization.

### MRI Data Acquisition

MR data were acquired using a Siemens 7T Magnetom system (Siemens ealthcare, Erlangen, Germany) with a 32-channel head coil. Blood oxygenation level dependent (BOLD) fMRI was acquired with a T2*-weighted multi-slice gradient echo planar imaging (EPI). True axial slices were positioned for optimal coverage of the left and right anatomical hand knob (Yousry et al. 1997) (TR/TE: 1500/25 ms, resolution: 1.2 mm isotropic, 22 axial slices, flip angle: 90°; GRAPPA factor: 2; anterior-posterior phase-encoding direction; 391 measurements). Magnetization prepared rapid gradient echo (MPRAGE) structural MRI data were acquired to facilitate BOLD EPI slice placement and for cortical surface reconstruction (TR/TE: 2200/2.82 ms, isotropic resolution: 1.0 mm, GRAPPA factor = 2). An additional gradient echo BOLD EPI acquisition of 4 volumes was acquired using posterior-anterior phase-encoding direction for distortion correction.

### fMRI Behavioral Task

During the fMRI acquisitions, participants performed a total of 10 runs of the motor task (5 runs per MRI session). Participants were laid supine with their right forearm supported against their right hip and their elbow supported by a foam pad, while wearing the data glove. Participants viewed instructions via a mirror mounted on the transmit coil and a projector screen mounted at the end of the bore. Each movement block comprised of a 3 s instruction screen (“Prepare to Move”), a 3 s video of the movement to be produced, and a 1 s further instruction screen (“Move”), followed by 5 movement trials, each comprising 1.6 s of movement (green expanding/contracting bar), followed by a 0.4 s rest period (red static bar). Each movement block was 17 s. In addition to the movement blocks, 8 rest blocks were included in each task run; rest blocks were of equivalent duration to movement blocks and comprised of a 3 s instruction screen (“Rest”), a 3 s video of a static resting hand, and a 1 s further instruction screen (“Rest”), followed by the same period of expanding and contracting bar visual stimuli as the fMRI movement blocks. Rest blocks were positioned randomly in each run, excluding self-adjacency.

### Structural MRI Data Preprocessing

MPRAGE data were subject to reorientation, bias-field correction and brain extraction using the FMRIB Software Library (FSL) fsl_anat tool (Zhang et al. 2001; Smith, 2002; Jenkinson et al. 2012) prior to cortical surface reconstruction using FreeSurfer Version 5.3.0 (Dale et al. 1999; Fischl et al. 2001).

### fMRI Data Analysis

#### fMRI Preprocessing and General Linear Modeling

fMRI data were subject to standard preprocessing, including motion correction with MCFLIRT (Jenkinson et al. 2002), brain extraction using BET (Smith, 2002), and high pass temporal filtering (100 s threshold). fMRI data were not subject to spatial smoothing. All fMRI data were subject to manual independent components analysis denoising (Griffanti et al. 2017). Distortion correction was undertaken using FSL Topup to estimate a fieldmap image for use in FSL FUGUE (Glasser et al. 2013). Undistorted BOLD EPI data were coregistered with structural MPRAGE data using Boundary-Based-Registration from FMRIB’s Linear Registration Tool implemented in epi_reg (Jenkinson and Smith 2001; Jenkinson et al. 2002; Greve and Fischl 2009). Example fMRI timeseries from a single voxel located in the anatomical hand knob is presented for four participants on a single session in Supplementary Figure S15.

For each participant and each fMRI run, fMRI data were analyzed using a first-level general linear modeling (GLM) approach implemented in FSL FEAT (Jenkinson et al. 2012) using the FMRIB Improved Linear Model to estimate time series autocorrelation and prewhiten each voxel. Each of the 26 movements was modeled with a separate boxcar regressor with gamma-HRF convolution and its temporal derivative, giving a total of 52 regressors. Parameter estimates were calculated, contrasting each movement type against the rest condition; these voxel-wise maps and an estimate of the residuals from the GLM were resampled into the respective participants’ structural space and used in subsequent RSA.

#### fMRI Multivariate Noise Normalization

In order to account for the spatial structure of the noise inherent to fMRI data, spatial prewhitening of the parameter estimates from each participant and each fMRI task run was conducted. The residuals (R) from the first-level GLM analysis provided an estimate of data not fit by the model regressors across voxels (V) and time (T), from which a V × V covariance matrix ($\hat{\epsilon}$) can estimate the noise structure across voxels (Equation (1)) (Walther et al. 2016):

(1) \begin{equation*} \widehat\Sigma=\frac{1}{T}{R}^TR \end{equation*}

The noise covariance structure was combined with the voxel-wise parameter estimates (*P*) for a given movement type (*k*) to generate a spatially prewhitened parameter estimate (${P}_k^{/\!\!\leftarrow }$: Equation (2)):

(2) \begin{equation*} P_k^{\ast}={P_k}{\widehat{\Sigma}^{-\frac{1}{2}}} \end{equation*}

#### fMRI Surface-Based Searchlight RSA

A surface-based RSA searchlight approach was used to identify regions in which the multivariate pattern of BOLD activity mirrored the kinematic and categorical models. This surface-based analysis constrained the voxels under consideration in each searchlight to the gray matter and prevented the issue of sampling of voxels that span a sulcus in a single searchlight, which is inherent to volumetric approaches (Oosterhof et al. 2011). A searchlight was constructed at the centre of each vertex within the individual participants’ anatomical cortical surface region corresponding to the field of view of their task fMRI data (Supplementary Figure S7). Each searchlight had a diameter of 10 mm. The region of interest of each searchlight was projected from two-dimensional surface to three-dimensional volumetric space using the Connectome Workbench Tool (Glasser et al. 2013), masked by a FMRIB Automatic Segmentation Tool gray matter map (Zhang et al. 2001) and a mask excluding voxels spanning across sulci in the FreeSurfer reconstruction to improve spatial specificity. Spatially prewhitened parameter estimates were extracted from the resulting volumetric region corresponding to each searchlight.

#### fMRI Cross-Validated Distance Measures

Within each searchlight the similarity between each of the spatially prewhitened voxel-wise parameter estimates corresponding to each of the 26 different movement types was calculated using a cross-validated approach to avoid the possibility of overfitting the data (Diedrichsen and Kriegeskorte 2017; Haynes, 2015). In each iteration, the parameter estimate maps from one fMRI task run was assigned to fold A and the parameter estimate maps from the remaining nine task fMRI runs were assigned to fold B; squared Euclidean distances were calculated between all possible pairs of the 26 movement parameter estimate maps across these two-folds (Equation (3)). Distance measures were calculated across all possible pairs of cross-validation folds and averaged (Walther et al. 2016). The use of spatially prewhitened parameter estimate combined with the cross-validation approach yielded cross-validated Mahalanobis distance representational dissimilarity matrices (RDMs) comparing each of the activation patterns across all possible pairings of the 26 movements. For example, calculation of the distance between movement k and movement l in one iteration:

(3) \begin{equation*} {d}^2_{\mathrm{Crossvalidated \ Mahalanobis}}\left({P}_k^{\ast },{P}_l^{\ast}\right)=\left({P}_k^{\ast }-{P}_l^{\ast}\right)_A{\left({P}_k^{\ast}-{P}_l^{\ast}\right)}_B^T \end{equation*}.

**Figure 2:**
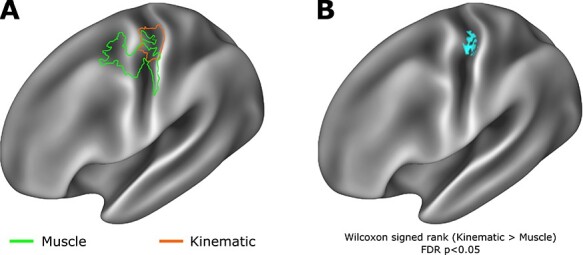
Kinematic and muscle models show evidence of distinct spatial encoding in primary motor cortex. (*A*) Outline of supra-threshold RSA results presented in Fig. 1 reveal overlapping but distinct encoding of muscle and kinematic information, with muscle information encoding in more rostral regions of Brodmann areas 4 and 6, while kinematic information is encoded in more caudal regions of primary motor cortex, including Brodmann areas 4 and 3a. (*B*) A Wilcoxon signed-rank test calculated on Spearman’s *ρ* values across the muscle and kinematic spatial searchlights revealed a region at the border of Brodmann areas 4 and 3a in which kinematic information showed significantly greater encoding than the muscle model (Statistical maps subject to FDR correction *α* = 0*.*05).

The correspondence between the fMRI-derived RDM in each searchlight and the candidate group average kinematic, muscle, and ethological models was assessed using a Spearman’s rank correlation, with the resulting *ρ* (rho) value plotted in each searchlight’s central vertex on the cortical surface. Spearman’s *ρ* was selected because it is rank based, and therefore does not require assumptions regarding the distributions of the input variables: this allows for the comparison of models derived from different source data. For statistical inference, a fixed effects randomization test (Nili et al. 2014) was applied on the individual participant level: correlations using 10 000 condition-label randomizations were undertaken in each searchlight. From each of the permutations, the spatial peak *ρ-*value (rho) was extracted from across the cortical surface, forming a maximum accuracy distribution from which an omnibus threshold (*α* = 0*.*01) was extracted. The resulting thresholded *ρ*-value surface maps for each participant were resampled onto the Human Connectome Project 32k surface (S1200*.*L.pial.MSMAll.32k_fs_LR.surf.gii), binarized and used to form a heatmap corresponding to the spatial distribution of each model fit across participants. In light of the interest in contrasting the kinematic and muscle models, a comparison of the corresponding unthresholded Spearman’s *ρ* cortical surface maps was undertaken using a Wilcoxon signed-rank test (one-sided), subject to FDR correction (*α* = 0*.*05) (Fig. 2).

### fMRI Motion Considerations

Variability in the magnitude of fMRI motion across different movement conditions has the potential to influence the observed pattern of results. The potential for noise induced by participant motion was mitigated in a number of ways. First, all data were subject to ICA denoising to remove any characteristic motion artifacts (Griffanti et al. 2017). Second, the multivariate analysis of fMRI data employed herein used spatial prewhitening of the parameter estimates to account for voxel-wise variability in order to not down-weight voxels with high error variance and to account for noise covariance between voxels (Walther et al. 2016). Finally, DVARS values were calculated for each fMRI timeseries (D: temporal derivative of time courses, VARS: root mean squares variance over voxels). These values quantify for each frame of an fMRI acquisition the magnitude of signal intensity change in volume N compared with volume N-1, as per the following formula:

(4) \begin{equation*} \mathrm{DV\,ARS} (\varDelta I)_i = \sqrt{\left\langle [{I}_i\big(\stackrel{\rightarrow}{x}\big)-{I}_{i-1}\stackrel{\rightarrow}{x}]^2\right\rangle} \end{equation*}

where *I_i_* is image intensity at locus *^-!^x* on frame *i*; angle brackets denote the spatial average over the whole brain (Power et al. 2012). DVARS are able to quantify corruption of fMRI acqusition due to head motion. DVARS values were extracted for volumes corresponding to each of the 26 hand movements for all participants; the resulting distribution of DVARS values is presented in Supplementary Figure S13. The profiles of very limited motion across participants during each session of around 10 min in duration also demonstrate high quality data acquisition (Supplementary Figure S14).

### MEG Data Acquisition

MEG signals were measured continuously at 1200 Hz during the motor task using a whole-head 275-channel axial gradiometer CTF MEG system (CTF, Vancouver, Canada) located inside a magnetically shielded room. An additional 29 reference channels were recorded for noise cancelation purposes and the primary sensors were analyzed as synthetic third-order gradiometers (Vrba and Robinson 2001). Three electromagnetic coils were placed on three fiduciary locations (nasion, left and right preauricular) and their position relative to the MEG sensors were recorded continuously during each experimental block. The head surface and fiducial locations were digitized using an ANT Xensor digitizer (ANT Neuro, Enschede, Netherlands) prior to the MEG recording.

### MEG Behavioral Task

During the MEG data acquisitions participants performed a total of 10 runs of the motor task (5 runs per MEG session). Participants were sitting upright with their right forearm and elbow supported on a foam armrest, while wearing the data glove. Participants viewed instructions on a back-projected screen in front of them from a projector mounted outside the shielded room. Each movement block comprised of a 2 s period with a central fixation cross, a 3 s video of the movement to be produced, and a 1 s instruction screen (“Prepare to Move”) followed by five movement trials, each comprising 1.6 s of movement (green expanding/contracting bar), followed by a 0.8 s rest period (red static bar). Each movement block was 18 s. The order of movement blocks was randomized within each task run; each movement was presented once per task run.

### Data Glove Movement Onset Detection: MEG Sessions

The 14 channels of data glove recordings collected during the MEG sessions were synchronized with the MEG acquisitions. Epoched data glove recordings were subject to onset segmentation using an adaptive threshold (activity duration threshold: 200 ms, no smoothing) (Hooman Sedghamiz: Matlab File Exchange: Automatic Activity Detection in Noisy Signals using Hilbert Transform.). A conservative estimate of movement onset was derived by taking the earliest signal onset detected across the 14 data glove channels for each movement trial (Supplementary Figure S9). The resulting movement onset time was used to epoch MEG data in further analysis.

### MEG Data Analysis

#### MEG Preprocessing

Each participant’s head shape was digitized using Xensor digitizer software (ANT software BV, Enschede, The Netherlands). All MEG analysis was conducted using the Fieldtrip toolbox for EEG/MEG-analysis (Oostenveld et al. 2011) (Donders Institute for Brain, Cognition and Behaviour, Radboud University Nijmegen, The Netherlands. See http://www.ru.nl/neuroimaging/fieldtrip). Coregistration was performed in a two stage process: first the fiducial locations were marked on the T1 structural for that participant; the head digitization data was then used to align the data with the MRI, subject to manual adjustment. Alignment was undertaken independently for data from the two MEG sessions.

Data from each movement type were epoched from the 10 task runs and concatenated into a new dataset containing 10 blocks, each containing 5 movement trials. The fixation cross and movement trials were epoched from the overall block. The movement trials were defined relative to the data glove defined movement onset time (movement trial time: 2 s; preonset time: 0.5 s, postonset time: 1.5 s). The fixation cross period was used as a baseline for the 5 movement trials within each movement block. A high pass filter of 1 Hz and a low pass filter of 100 Hz were applied. MEG analyses were conducted across four frequency bands: alpha (7–14 Hz), beta (15–30 Hz) and gamma (30–100 Hz), and broad band (7–100 Hz). All of the movement trials for a given movement type were concatenated across the 10 task runs, creating a dataset comprising 50 repeats of a movement. At this point, the data was visually inspected and those trials containing artefacts were removed from further analysis up to a maximum of 10 trials, such that the minimum number of movements trials per movement included in further analysis was 40.

#### MEG Source Reconstruction

In order to reconstruct oscillatory activity at brain locations directly comparable across participants, the individual anatomical MRI was nonlinearly warped to the MNI MRI template. The MNI template was divided into a 10 mm isotropic grid and the inverse of the previously calculated nonlinear warp was used to warp the template grid into the anatomical space of each participant. Sensor leadfields were calculated using a semirealistic volume conduction model based on the individual anatomy (Nolte, 2003). The temporal evolution of source activation at each location in the brain was estimated using a linearly constrained minimum variance (LCMV) beam-former algorithm (Veen et al. 1997) with the optimal dipole orientation at each voxel estimated using singular value decomposition. Virtual sensors were then reconstructed from all 3294 voxels by multiplying the sensor level data by the corresponding set of optimized weights. At this stage, data were subject to multivariate noise normalization (Guggenmos et al. 2018; Ledoit and Wolf 2004), we calculated the error covariance matrix at sensor level and then used this combined with the filters from the LCMV to create the virtual sensor data. This means that sensors with more noise would be down-weighted compared to those with less noise. At this stage, the data were also down-sampled to 600 Hz to reduce computational cost.

#### MEG Temporal RSA

The MEG data were split to produce 10 partitions and then averaged within each partition to perform a cross-validated representational similarity analysis to avoid the possibility of overfitting the data (Diedrichsen and Kriegeskorte 2017; Haynes, 2015). RSA was performed across time using a sliding time window with a width of 20 ms and a time step of 5 ms creating 396 time windows across 2 s of the movement trial (0.5 s rest, 1.5 s movement). After selecting virtual sensors within the left hemisphere motor region of the AAL atlas (Tzourio-Mazoyer et al. 2002) (Precentral L, 31 sources; Supplementary Figure S8), the frequency-filtered MEG signal measured during each movement type was compared using a cross-validated approach within each time width. In each iteration, the signals from one MEG data partition were assigned to fold A, and the signals from the remaining nine partitions were assigned to fold B; squared Euclidean distances were calculated between all possible pairs of the 26 signals across the two-folds and averaged (Walther et al. 2016). The use of multivariate noise normalization to account for spatial autocorrelation in the MEG signal yielded subject-wise cross-validated Mahalanobis distance RDMs comparing the alpha-, beta-, or gamma-band signal in the motor Region of Interest (ROI) across all possible pairings of the 26 movements (Guggenmos et al. 2018).

Participant-level motor ROI RDMs were averaged in order to perform a fixed-effects analysis. The correspondence between the MEG-derived RDMs and the candidate group average kinematic, muscle, and ethological models across time was assessed using a Spearman’s rank correlation, with the resulting *ρ* (rho) values plotted for each time window. As in the fMRI analysis, a rank-based correlation was used to allow for the comparison of models originating from different source data without making assumptions about the distribution of values within these models. In light of the interest in contrasting the kinematic and muscle models, these were each assessed in a partial correlation to discount the contribution of the other. Randomization testing was used for statistical inference (Nichols and Holmes 2002), whereby candidate model RDMs were shuffled 1000 times and time-resolved correlation coefficients were recomputed in order to estimate an empirical null distribution. *P*-values were calculated using a cluster thresholding approach across time. To correct for multiple comparisons, the cluster-forming threshold was set to *P <* 0*.*01 and clusters in the correlation time-courses corresponding to each candidate model were thresholded against the maximal cluster distribution (*α* = 0*.*001).

To assess the maximal correlation possible with our data, each participant’s RDM was correlated with the average cross-subject RDM; the correlations were then averaged to obtain an upper bound of the noise ceiling (Nili et al. 2014).

#### MEG: Action Observation Analysis

MEG data from the period of action observation during the instruction video preceding each movement block were epoched using the same approach as the MEG data recorded during movement. The fixation cross and action observation trials were epoched from the overall block. The action observation trial was defined relative to the video stimulus onset time (preonset time: 0.5 s, postonset time: 3.0 s). The fixation cross period was used as a baseline for the action observation period. Temporal RSAs were conducted using the same approach as the MEG movement data, as described above.

#### MEG Motion Considerations

MEG analysis included multivariate noise normalization to account partially for the effects of motion, where each channel is normalized by an estimate of error covariance across different sensors; this process has been demonstrated to substantially improve multivariate analyses of MEG data (Guggenmos et al. 2018). Motion parameters for all MEG acquisitions were extracted and analyzed to rule out the possibility of excessive head motion as a potential driving force behind any observed patterns of brain activity. Rotational and translational displacement for each participant and each experimental session are presented in Supplementary Figure S11. In addition, the motion parameters during each movement block were extracted and the resulting distribution is presented across the 26 different movement types (Supplementary Figure S12). The profiles of motion across participants demonstrate a high quality data acquisition.

### Electromyography with MEG

EMG data were acquired simultaneously with MEG data. Three surface EMG electrodes were attached to the right hand underneath the data glove, positioned on APB, FDI, and ADM. The area under the electrodes was exfoliated and cleaned with alcohol prior to data acquisition. EMG signals were recorded at 1200 Hz.

EMG data were initially subject to a bandpass filter (20–1000 Hz) and a notch filter (50 Hz). EMG data were epoched and baselined alongside the MEG data. Epoched EMG data were subject to manual artifact rejection. Signals from the three electrodes during each epoch were independently subject to a Hilbert transform and smoothing (5 ms smoothing window) prior to activity onset segmentation using an adaptive threshold (activity duration threshold: 200 ms) (Hooman Sedghamiz: Matlab File Exchange: Automatic Activity Detection in Noisy Signals using Hilbert Transform). A conservative estimate of muscle activity onset was derived by taking the earliest signal onset detected across the three EMG channels for each movement trial; any trial in which the onset estimate from the EMG and data glove activity recorded during MEG showed a discrepancy of *>±*100 ms was excluded. Due to constraints of electrode placement alongside the kinematic data glove, measures of activity onset were not robustly measured in all participants. EMG onset data are presented in order to validate the data glove measures of movement onset, which have been used to epoch the MEG data (Fig. 1 and Supplementary Figure S10).

## Results

We first used high-resolution fMRI data to perform a cross-validated cortical surface-based searchlight RSA to find evidence for the spatial encoding of kinematic information during movement. In each participant and each cortical searchlight, the unsmoothed pattern of fMRI activity during movement was used to construct a RDM (Nili et al. 2014). The RDM was compared to group average kinematic or muscle models (3D graphics 1 and 2), and a theoretical ethological action model, resulting in representational similarity cortical surface maps of Spearman’s *ρ* values for each participant and model. Spearman’s *ρ* surface maps for each model were subject to an omnibus threshold (*α* = 0*.*01) and used to construct a cross-participant heatmap. This analysis assessed where the relative dissimilarities in the kinematic, muscle and ethological actions across the different hand movements were mirrored by the relative differences in the pattern of fMRI activity elicited by performing the same movements.

For the kinematic model, the searchlight revealed a strong and very consistent representational similarity in the contralateral precentral region of the anatomical hand-knob (Yousry et al. 1997) across participants (Fig. 1, top row). Specifically, the fMRI searchlight results revealed the consistent encoding of the kinematic information in Brodmann area 4 during the production of hand movements across participants (Table 2) (Glasser et al. 2016). This means that the same differences we observed in the kinematics of our 26 movements were also observed in differences in the patterns of BOLD activity measured in caudal M1 during movement production.

**Table 2:** Outline of peak anatomical correspondence between movement models and fMRI calculated using across participant cortical heatmaps

Model	Peak heatmap overlap (Participants)	Peak vertex	Anatomical location
Kinematic	10	8053/5378	Brodmann area 4
Muscle	10	5070	Brodmann area 4
		8015/8044	Brodmann area 3b
Ethological	8	8070	Area 3b

Peak regions calculated as centre of gravity of areas of peak overlap; peaks separated by a minimum of 20 mm. Vertex positions and anatomical definitions are based on HCP S1200 32k release (Glasser et al. 2016).

Inspection of the single-subject cortical searchlight results for the kinematic model highlights the consistent and spatially limited correspondence of the kinematic model and fMRI data at the level of individual participants in contralateral M1 (Fig. 2*A*). In the contralateral hemisphere, the peak spatial overlap in the encoding of kinematic information across participants was observed in Brodmann areas 4 and 3a; other regions to reach significance at the level of individual participant searchlight analyses, but were not observed consistently across the entire group, include Brodmann area 3a, Brodmann areas 2, 3b, and Brodmann area (Supplementary Figure S3). A highly comparable result was also observed using the group average kinematic model constructed from the data glove recordings made in the behavioral testing session (Supplementary Figure S19), highlighting the applicability of this result to real-world hand use in an upright sitting position. No such consistent representational similarity was observed in the corresponding searchlight of movement-related activity in the ipsilateral hemisphere at the group level; however, at the level of individual participants, significant encoding was observed in greater than three participants included Brodmann areas 4, 3a, and 6 (Fig. 2B and Supplementary Figure S19B).

Equivalent spatial searchlight analyses for the muscle model also revealed supra-threshold activity consistent with encoding in the precentral region of the anatomical hand knob (Fig. 1, bottom row). The muscle model shared representational structure with patterns of brain activity in more rostral and ventral regions compared with the kinematic model, including both areas of Brodmann areas 4 and 6, as well as areas of Brodmann area 3b. This pattern showed less spatial consistency across participants (Figure 1 and Supplementary S6). This means that differences in the pattern of muscle activity measured across different movements were mirrored by differences in the associated patterns of BOLD activity in rostral M1 during movement production.

In light of the interest in contrasting the kinematic and muscle models (Leo et al. 2016), a Wilcoxon signed-rank test (one-sided) was used to compare the vertex-wise *ρ* maps of these two models, which demonstrated the superior fit of the group average kinematic model in comparison to the group average muscle model in a localized region principally corresponding to Brodmann areas 4 and 3a (Nili et al. 2014) (Fig. 2).

The ethological action model (Supplementary Figure S18A) revealed more limited evidence of consistent cortical encoding across participants, centered on somatosensory cortex in the postcentral gyrus; specifically, Brodmann area 3b (Supplementary Figure S18B).

High field fMRI data analyzed at the level of individual subjects offered detailed spatial resolution, revealing distinct encoding of kinematic and muscle information in different areas of the hand knob region of M1.

However, fMRI offers relatively poor temporal resolution to understand the point in time at which the kinematic and muscle models match the pattern of brain activity in M1. The boundary between motor and somatosensory cortex is increasingly blurred by evidence of sensory processing in M1 (Hatsopoulos and Suminski 2011) and motor modulation of sensory afferents (Lee et al. 2008). The encoding of muscle and kinematic information observed from patterns of fMRI activity may result from top-down control of motor function, or from bottom-up proprioceptive information passed back to M1 and S1. In order to dissociate the driving force behind the spatial model fit observed in the fMRI data, a temporal RSA of MEG data was used to identify the point during movement preparation or execution at which kinematic and muscle information is encoded in the M1.

A cross-validated fixed-effects RSA was applied, comparing a group average of the kinematic and muscle models to the pattern of alpha (7–14 Hz), beta (15–30 Hz), gamma (30–100 Hz) and broad (5-100 Hz) band MEG brain activity in M1 (Supplementary Figure S8) in 20 ms sliding windows during movement preparation and execution. The ethological action model was assessed in equivalent analyses. In light of the interest in contrasting the group average kinematic and muscle models, these models were assessed using a Spearman’s correlation, as well as in a partial correlation to discount the contribution of the other (Supplementary Figure S21).

**Figure 3:**
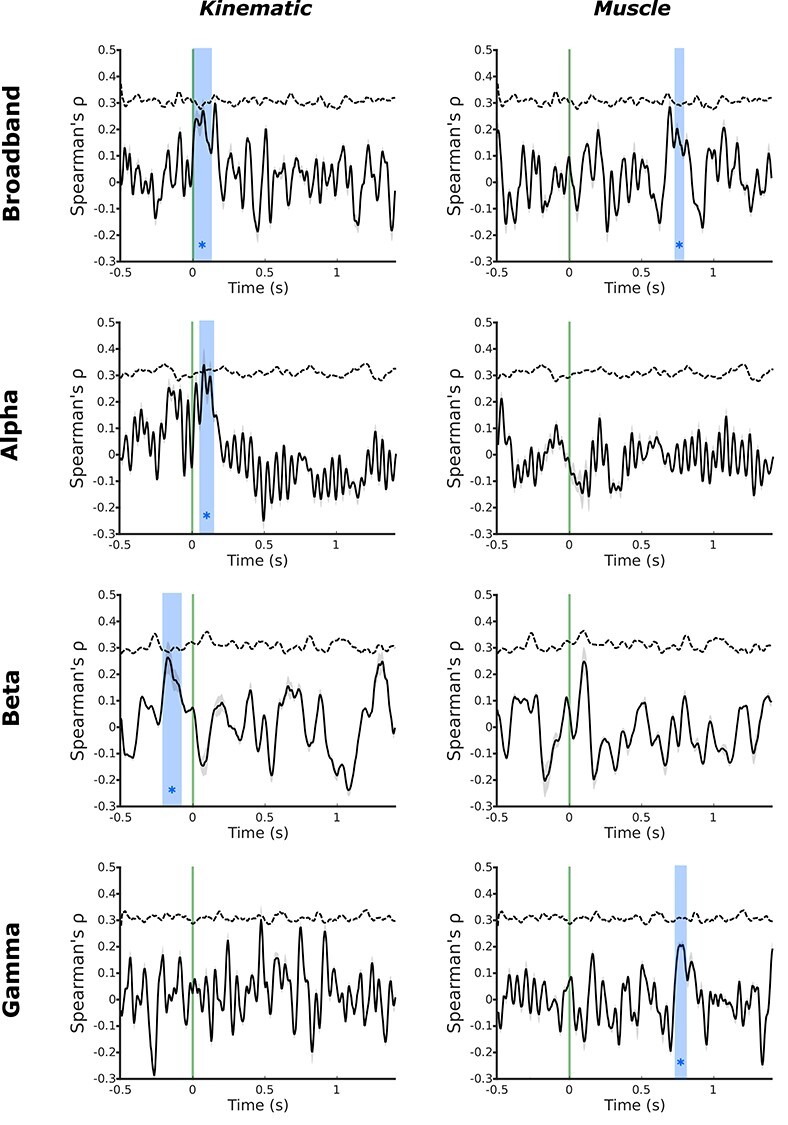
MEG temporal RSA searchlight in motor cortex reveals distinct encoding of kinematic and muscle information. Temporal MEG searchlight analysis of the broadband MEG signal revealed encoding of kinematic information around the time of movement onset (5–120 ms), contrasted against much later encoding of muscle information 735–785 ms after movement onset. Decomposition of the MEG signal into alpha, beta, and gamma frequencies revealed distinct encoding of the group average kinematic and muscle models across bands. The kinematic model showed significant encoding in the alpha band after movement onset (55–135 ms) and the beta band prior to movement onset (−210 to −90 ms). In contrast, the muscle model showed significant encoding in the gamma band substantially after movement onset (735–795 ms). Green line—movement onset defined by the data glove; blue regions—significant peaks in representational similarity between MEG data and the model (1000 shuffled permutations of candidate model RDMs; cluster-forming threshold: *P <* 0*.*01; maximal cluster distribution (*α* = 001); dashed line—correlation noise ceiling.

Temporal MEG searchlight analysis revealed distinct temporal encoding of the kinematic and muscle models in the alpha, beta, and gamma frequencies. The kinematic model showed significant encoding in the alpha band immediately after movement onset (55–135 ms). In the beta band, the kinematic model mirrored the pattern of brain activity in a significant peak from before movement onset (−210 to −90 ms). This means that the same differences that we observe in the kinematics of the 26 different movements under study are also observed in the oscillatory activity of motor cortex up to 200 ms before these movements even begin. Specifically, before a movement is initiated, information about the upcoming kinematics is encoded in the beta oscillations recorded from primary motor cortex. In contrast, the muscle model showed significant encoding in neural activity substantially after movement initiation, which originated from a temporal correspondence with information encoded in the gamma band (735 to 795 ms relative to movement onset) (Fig. 3). This means that over 700 ms after movements were initiated, differences in the pattern of high frequency oscillations in motor cortex mirrored differences in muscle activity across the 26 different movements.

An analogous MEG temporal searchlight analysis during action observation revealed evidence of a correspondence between the kinematic model and brain activity during the movement videos preceding each movement block (Supplementary Figure S4). During action observation a correspondence between the MEG signal and kinematic model was observed from 220–255 to 890–955 ms in the alpha band, 705–735 ms in the beta band, and 545–560 ms in the gamma band, relative to stimulus onset. No peaks in any frequency band were observed for the muscle model or the ethological action model during the period of action observation.

## Discussion

Taken together, the MEG and fMRI results presented here strongly implicate the distinct spatial and temporal encoding of kinematic and muscle information in M1. Specifically, fMRI data suggest that kinematic information is represented more caudally in M1, in Brodmann areas 4 and 3a. Complementary MEG data suggested that kinematic information is encoded prior to and immediately following movement onset in oscillatory neuronal activity in alpha and beta frequencies (Fig. 3). In other words, the relative differences in the kinematic structure of a range of different hand movements is encoded in M1 up to 210 ms before the onset of movement can be detected in the hand.

In contrast, the muscle-based movement model was encoded in more rostral regions of M1, including Brodmann areas 4 and 6 (Figs. 1 and 3). Temporally, the muscle model was encoded much later in the cycle of movement, starting at 735 ms after movement onset in the gamma frequency (Fig. 3).

These results present strong new evidence in our understanding of movement encoding in M1. They suggest that kinematic features of movements are encoded immediately prior to and during the initiation of a movement, consistent with a role for this organization’s structure in top-down motor control. In contrast, brain activity that mirrors EMG recordings of muscle activity was observed much later after movement onset in more anterior regions of motor cortex; suggestive of a role in bottom up signaling later during movement production.

The observation of distinct rostral and caudal representational structures in human M1 is in keeping with an extensive primate literature reporting markedly distinct connectivity profiles along this axis of M1 in non-human primates. Specifically, retrograde labelling studies have reported that the evolutionarily newer caudal region of M1 contains a very high density of cortico-motorneuronal cells (CM cells): those which make monosynaptic connections with motoneurons and are associated with highly skilled movements. In contrast, the evolutionarily older rostral M1 contains few, if any, CM cells, relying instead on integrative processes mediated via connections to interneurons in the spinal intermediate zone (Rathelot and Strick 2009).

The observed kinematic information encoding in caudal M1 is in keeping with the notion of this cortical region containing CM cells that facilitate specific muscle synergies (Cheney and Fetz 1985). The evolutionary development of this caudal M1 region has been specifically associated with the rise of manual dexterity in non-human primates: for example, the existence of large populations of CM cells with monosynaptic connections to motoneurons in the ventral horn of the spinal cord is a hallmark of the ability for independent finger use in the cebus monkey when compared to the squirrel monkey, which has a similar hand structure, but lacks direct cortico-motoneuronal projections (Bortoff and Strick 1993). These direct connections via CM cells are not present at birth, but rather develop during early life, and mirror patterns of enhanced dexterous function during infancy and childhood (Olivier et al. 1997).

In contrast to encoding of kinematic information in caudal M1, we observed encoding of muscle information in more rostral regions of M1 (Fig. 2). Lacking CM cells, rostral M1 has been associated with movement via pattern generators or motor primitives via connections to spinal interneurons. In cats, which exhibit only a rostral M1, electrical stimulation to motor cortex elicits movements restricted to very precise muscular anatomy (Nieoullon and Rispal-Padel 1976), rather than the patterns of complex movement observed in similar studies of non-human primates (Graziano, 2016). In addition, the inputs to rostral M1 differ from caudal M1: neurons responsive to deep muscle or joint sensory input are concentrated in rostral M1, while cutaneous sensory inputs are concentrated in caudal M1 (Rathelot and Strick 2006; Tanji and Wise 1981; Picard and Smith 1992).

Our results provide functional evidence for organizational and temporal differences in the previously described rostral and caudal divisions of M1. Caudal M1, with its direct motoneuronal projections, here showed evidence of encoding movement kinematics, prior to and immediately following movement onset, during the production hand movements. Rostral M1, with its strong deep muscle/joint sensory inputs, showed evidence for the encoding of muscle-based information derived from EMG recordings, which occurred 735–795 ms after movement onset, strongly consistent with bottom-up sensory signaling from deep joint and muscle receptors. This spatial and temporal dissociation of functional organization in M1 provides a unique insight into the cortical control of dexterous movements.

Information contained in the kinematic model showed temporally distinct correspondence to information contained in the alpha and beta bands of the MEG data. From 210 to 90 ms before movement is detected, the representational structure in the M1 beta band corresponds significantly to the representational similarity of the kinematics of the upcoming movement. In other words, even before a movement begins, beta oscillatory brain activity already differs depending on the kinematics of the upcoming movement.

Beta oscillations are observed at rest; it is well established that beta activity is suppressed immediately prior to and during movement: movement-related beta desynchronisation (MRBD), and then rebounds following movement cessation: postmovement beta rebound (Pfurtscheller and Lopes da Silva 1999). The magnitude of the reduction in beta-band power observed prior to movement onset in motor cortex has been shown previously to relate to the degree of uncertainty in the upcoming movement (Tzagarakis et al. 2010) or action anticipation (Denis et al. 2017). Previous comparisons of beta desychronisation made across kinematic and kinetic tasks concur: the strength of MRBD is correlated with the physical kinematic displacement of a given hand movement rather than the magnitude of muscle contraction (Nakayashiki et al. 2014). Similar patterns of desynchronisation are observed in alpha band activity, where ERD in M1 corresponds to increased activation in the region (Pfurtscheller and Lopes da Silva 1999), with postmotion event related synchronization in M1 (Ohara et al. 2000). The postmovement peak in kinematic information encoding in the alpha band was observed early after movement onset, during a window of time in which the magnitude of ERD continues to increase after movement has begun (Babiloni et al. 1999). Here we demonstrate that there is a link between information contained in the beta frequency in M1 before movement onset and the subsequent kinematics of hand movements (Figs. 1 and 3), suggesting that the encoding of an upcoming motor command in beta oscillatory activity is based on the kinematic outcome of the planned movement (Nakayashiki et al. 2014; Engel and Fries 2010).

The observed concurrence between the group average muscle model and patterns of brain activity measured by MEG occurred sometime after movement onset (735–795 ms, Fig. 3). An increase in the amplitude of gamma oscillations has previously been reported during motor execution: movement-related gamma synchronization (Cheyne et al. 2008; Nowak et al. 2018). Increased gamma frequency power is correlated with the size of a given movement, but their strength does not persist during isometric contraction. However, increases in gamma power in M1 are not observed in passive movement conditions, suggesting that gamma activity is not directly associated with muscle activity alone, but rather muscle activity associated with limb movement and the associated sensory feedback (Muthukumaraswamy, 2010).

Hand kinematics have previously been investigated in the context of human fMRI. Relative differences in target joint position at the end of a hand movement have been shown previously to mirror the relative differences in the fMRI signal in a broad region of sensorimotor cortex (Leo et al. 2016).

Additional work considering unidigit and multidigit flexion has demonstrated that patterns of M1 fMRI activity associated with such movements are better explained by kinematic models of digit couse than by competing muscle-based models (Ejaz et al. 2015). In the present study, we have used MEG and 7 T BOLD fMRI to fundamentally extend on these findings. Specifically in the context of fMRI, high spatial resolution fMRI data enabled us to reveal a spatial dissociation in muscle and kinematic information encoding in M1 along the rostro-caudal axis (Fig. 2). We have been able to pinpoint a region of caudal Brodmann area 4 in which kinematic information shows significantly greater encoding than muscle information (Yousry et al. 1997). Taken alongside evidence from MEG for a temporal dissociation of kinematic and muscle information during the movement cycle, these data strongly implicate kinematic organization structure in top-down control of hand movements.

The fMRI spatial searchlight analysis did not reveal evidence of consistent encoding of kinematic information in ipsilateral M1 across participants (Supplementary Figure S3). Previous fMRI studies provide evidence for the activation of ipsilateral M1 during the production of individual unidigit movements (Diedrichsen et al. 2013; Berlot et al. 2018) but not multidigit sequences of unidigit movements (Yokoi et al. 2018). The present study considered a broad array of naturalistic hand movements, engaging a wide variety of hand kinematics, involving simultaneous and/or sequential movement of different digits. It is possible that unlike sequences of unidigit movement, these more complex movements do not drive the circuits of ipsilateral M1 as unidigit movements do (Diedrichsen et al. 2013; Berlot et al. 2018).

Previous studies have made direct comparisons between muscle-based models and kinematic models, arguing for the latter as an organizing principle in the encoding of hand movements (Ejaz et al. 2015; Leo et al. 2016). As with previous studies, the present findings do not rule out the existence of muscle representations in M1, but rather support the existence of highly organized muscle representations structured around movement kinematics rather than muscle anatomy. The assertion perhaps explains the fractures and repetitions observed in muscle representations during the search for an M1 body map (Lemon, 1988).

Data glove recordings were used to accurately define the point of movement onset in order to epoch MEG trials relative to this point. This approach enabled us to make precise and accurate statements regarding the nature of information encoding in sensorimotor cortex before and after movement began. The onset signals measured from data glove recordings were validated against more limited concurrent EMG recordings during MEG. Onset detection from EMG showed broadly later onset detection times when compared against data glove recordings. The data glove recordings potentially provided a slightly more conservative (i.e. earlier) estimate of movement detection because of the limited muscle coverage feasible with surface EMG electrodes. In any case, even using the more conservative movement onset detection times from the data glove, we observe the encoding of kinematic information over 200 ms prior to movement, supporting the notion that the kinematics of an upcoming movement are relevant in motor execution in M1.

The ethological action model reported less consistent patterns of fMRI encoding, centered on the postcentral gyrus, consistent with activation in S1 (Supplementary Figure S18). The ethological action model also did not reveal any significant peak in the temporal representational analysis. It is possible that while at a coarse level, ethological maps exist in the primate cortex, the concept of ethological organization does not extend down to the fine-grain level of individual encoding of human hand movements; in other words, the broad motor reportoire of the human hand may not be encoded on the basis of the functional role of each movement. However, in the case of the primate, the coarser division of movements based on the functional role of the entire upper limb, including the hand (e.g. feeding and reaching), may play a role in the way the cortex is organized (Graziano et al. 2002). The observed patterns of postcentral activity may alternatively result from selective disinhibition of S1 by M1 during motor activity, though such direct cortico-cortical signaling remains speculative in the human brain (Lee et al. 2008, 2013; Choi et al. 2018).

Analysis of the action observation period of the MEG data preceding each movement block also provided some support for the kinematic encoding of information in M1 (Supplementary Figure S4). Previous MEG data acquired during action observation have demonstrated characteristic changes in M1 activity comparable to action execution (Hari et al. 1998). Analyses of event-related desynchronisation (ERD) in M1 during action observation have suggested a peak change in the mu frequency as the observed movement evolves (Tani et al. 2018). These observations are potentially consistent with the pattern of kinematic model fit observed in the alpha and beta band MEG data during action observation, when the trajectory of movement has become clear (Supplementary Figure S4). Additional work considering the encoding of kinematic information in oscillatory alpha band activity in M1 suggests that the observation of stimuli consistent with biological motion is sufficient to induce ERD in this frequency band (Meirovitch et al. 2015), potentially consistent with the notion that during observation of biological motion, M1 may encode kinematic information. Given the focus of the present study on movement production, the infrequent and brief exposure of participants to action observation stimuli during the fMRI experiment did not provide sufficient data to make firm inferences regarding the spatial encoding of kinematic information while movements were observed. Based on existing meta-analyses, one would expect that such kinematic information could be encoded across a broad network of brain regions known to exhibit motor mirror properties (Molenberghs et al. 2012).

The data presented in this study rely on complementary information acquired from BOLD fMRI and MEG, though the remit of this work does not extend to fusion of the two modalities. BOLD fMRI provides only an indirect measure of neuronal activity based on haemodynamic changes associated with the execution of a given task (Jezzard et al. 2001), which can be resolved with a relatively high degree of spatial specificity with 7 T imaging. In contrast, MEG reflects a more direct, temporally rich, measure of neuronal activity. While the origins of the measured signals differ, compelling recent evidence provides noncoincidental data to support the notion of shared information across MEG and fMRI measures of brain activity across a wide range of frequency bands (Hipp and Siegel 2015); similar correspondences have been reported from invasive electrocorticography data (Siero et al. 2014). However, the spatial component of MEG data must be inferred from mathematical modeling. Despite advances in the context of MEG source localization, this feature of MEG analysis limits the spatial specificity of the measured signals, which integrate information across relatively large tissue volumes in comparison with fMRI (Hall et al. 2014). It is therefore not possible to definitely colocalize the signals from MEG and fMRI data. Thus, the motor cortex MEG signal used in the temporal multivariate searchlight analysis could have been influenced by signals from adjacent somatosensory cortex; mu-rhythm activity has been shown to associate with sensorimotor BOLD activity (Yin et al. 2016). However, previous data from comparative MEG/fMRI studies has suggested a broad association of the sensorimotor alpha frequency signal with the BOLD activity in the postcentral gyrus, and the beta frequency with BOLD activity in the precentral gyrus (Salmelin and Hari 1994; Salmelin et al. 1995; Cheyne et al. 2003; Ritter et al. 2009), a similar gradient has been supported broadly by intracortical recordings from non-human primates (Jasper and Penfield, 1949; Rougeul et al. 1979). Here, we observe a premovement encoding of kinematic information in the beta frequency, and a similar peak immediately after movement onset in the alpha frequency (Fig. 3). It is therefore possible to speculate that the beta frequency encoding is more likely to represent precentral activity in motor cortex, which would again support the conclusion that kinematic information is involved in the top-down control of dexterous movement.

In this work, we apply a rich multimodal design with multivariate analysis to provide evidence for spatial and temporal dissociations of kinematic and muscle-based information in human M1 during hand movement. Mounting evidence for the encoding of complex kinematic information in M1 from this and other work continues to blur the boundary between primary somatosensory and primary motor cortex: even M1 neurons have been shown to rapidly consolidate sensory torque information across multiple joints (Pruszynski et al. 2011). The notion of kinematic representation in M1 immediately prior to movement initiation is compatible with recent evidence of the tight integration of information across the central sulcus (Arce-McShane et al. 2016), whereby S1 encodes the current body state, while M1 encodes the kinematics necessary to achieve the intended body state. Such a system of motor control would see kinematic information encoded prior to movement onset as a prediction for the future sensory inputs expected by S1 when a movement has been achieved (Adams et al. 2013).

## Supplementary Material

Supplementary_Materials_tgaa009
