# Supplementary material for: Spatially and Temporally Distinct Encoding of Muscle and Kinematic Information in Rostral and Caudal Primary Motor Cortex
Source: Cereb Cortex Commun. 2020 Apr 4;1(1):tgaa009. doi: 10.1093/texcom/tgaa009 (PMC7446240; doi:10.1093/texcom/tgaa009)
Supplement: Supplementary_Materials_tgaa009 [file supplementary_materials_tgaa009.zip › Supplementary_Materials_tgaa009/Supplementary.pdf]

Supplementary information: Spatially and temporally distinct encoding of muscle and kinematic information in rostral and caudal primary motor cortex.

Video S1: Compilation of instructional videos used at the beginning of movement blocks in all testing sessions. Movement labels are provided for reference only; labels were not included during the task (VideoS1.mov).

Video S2: Visualisation of multidimensional scaling of group average kinematic model constructed across participants and sessions. (VideoS2.mov).

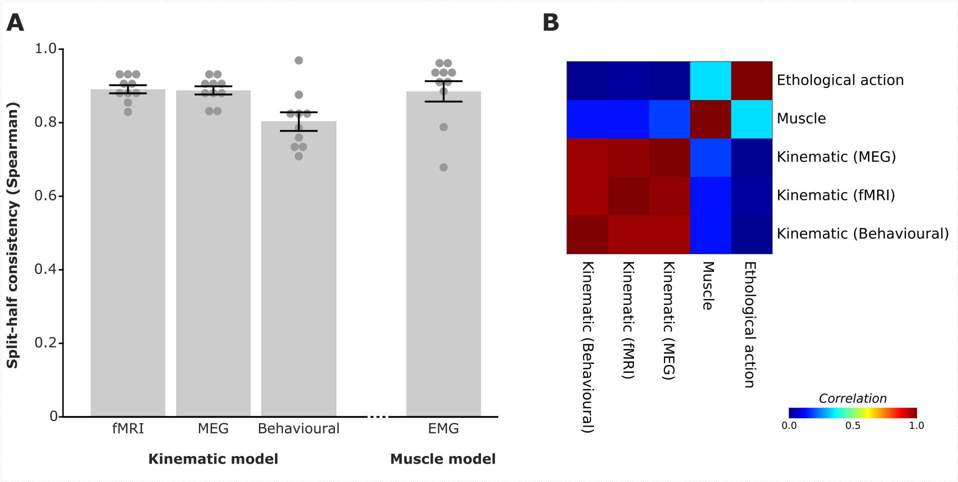

Figure S1: (A.) Data-driven kinematic models constructed for each participant and each session type exhibit strong split-half and inter-session consistency. Muscle model reproducibility data also presented. (B.) Strong between-model consistency across kinematic models calculated from data across fMRI, MEG, and behavioural recording sessions; limited shared information across kinematic and muscle models. Ethological action model resulted presented in Figure S18.

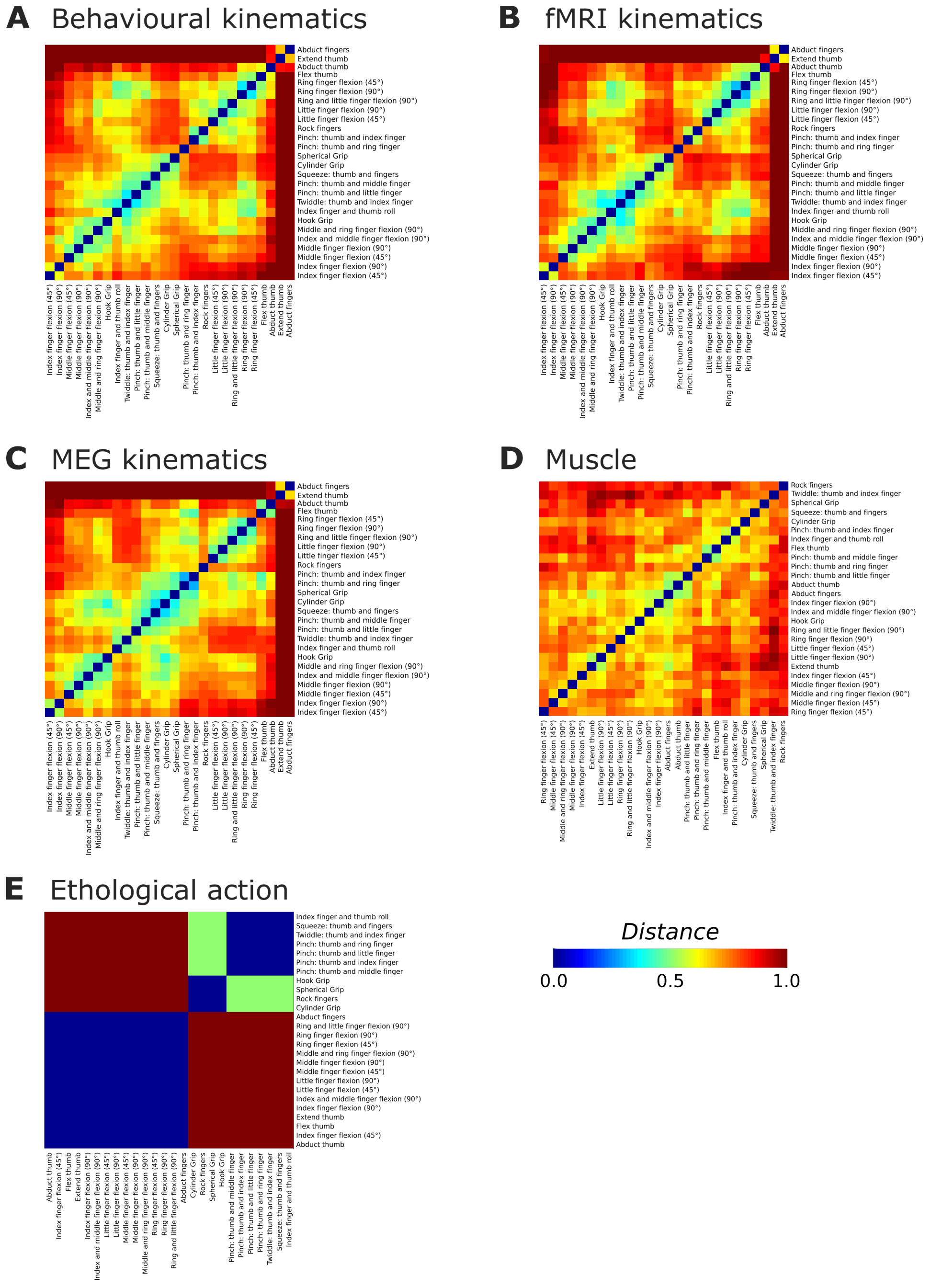

Figure S2: The group average kinematic model across participants for each recording session type, the group average muscle model derived from an independent cohort of participants, and the categorical ethological action model.

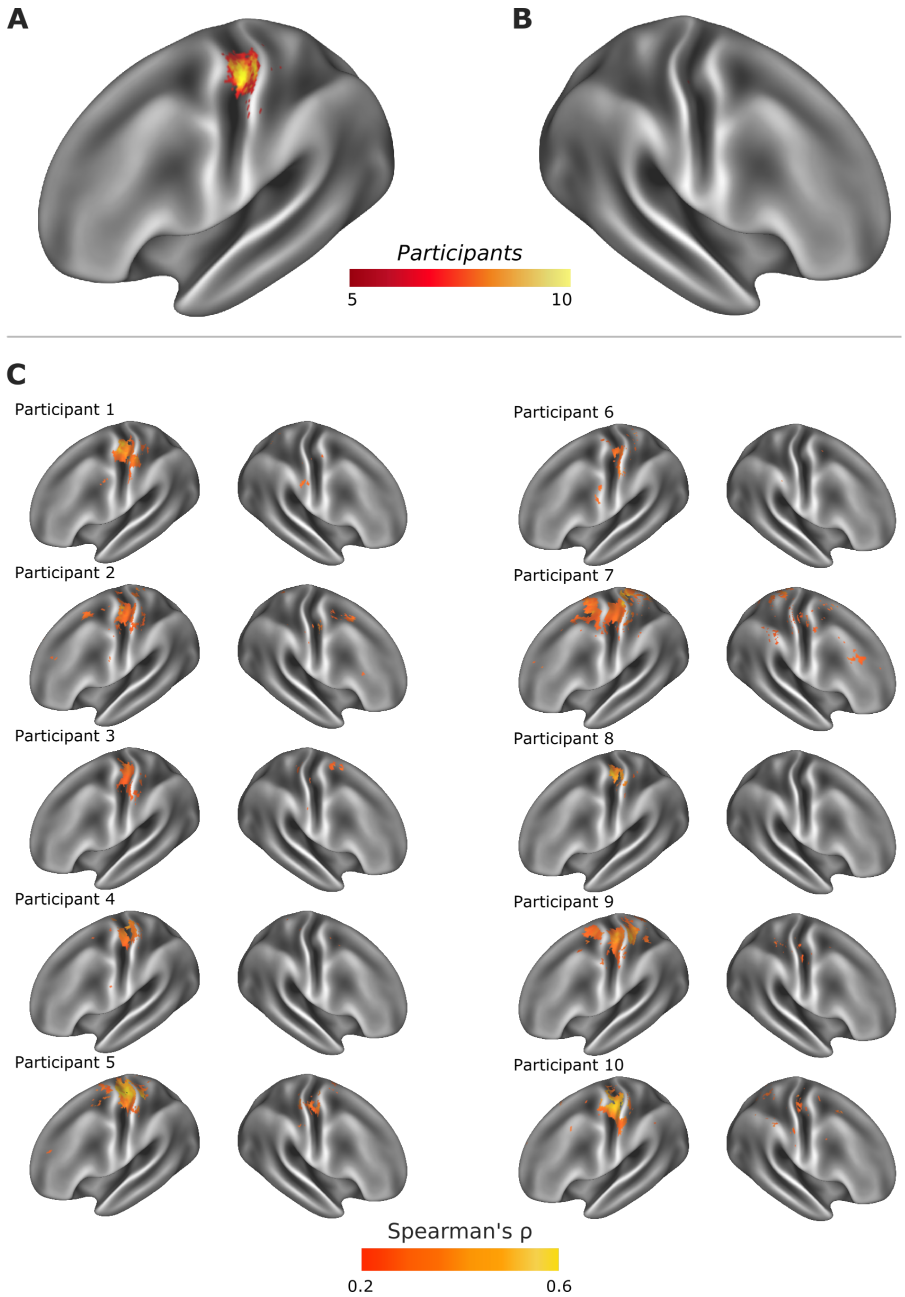

Figure S3: Single participant fMRI representational similarity analysis cortical searchlights using individual kinematic models of hand movement. Cortical heatmaps of the left (A) and right (B) hemisphere, show consistent encoding of kinematic information in the left motor cortex, contralateral to movement. Heatmaps were constructed from individually thresholded cortical searchlights for each participant, derived using their own kinematic model (C) (Omnibus threshold, $\alpha = 0.01$, maximum accuracy distribution calculated from peak correlation value across 10,000 searchlight permutations with label-switching).

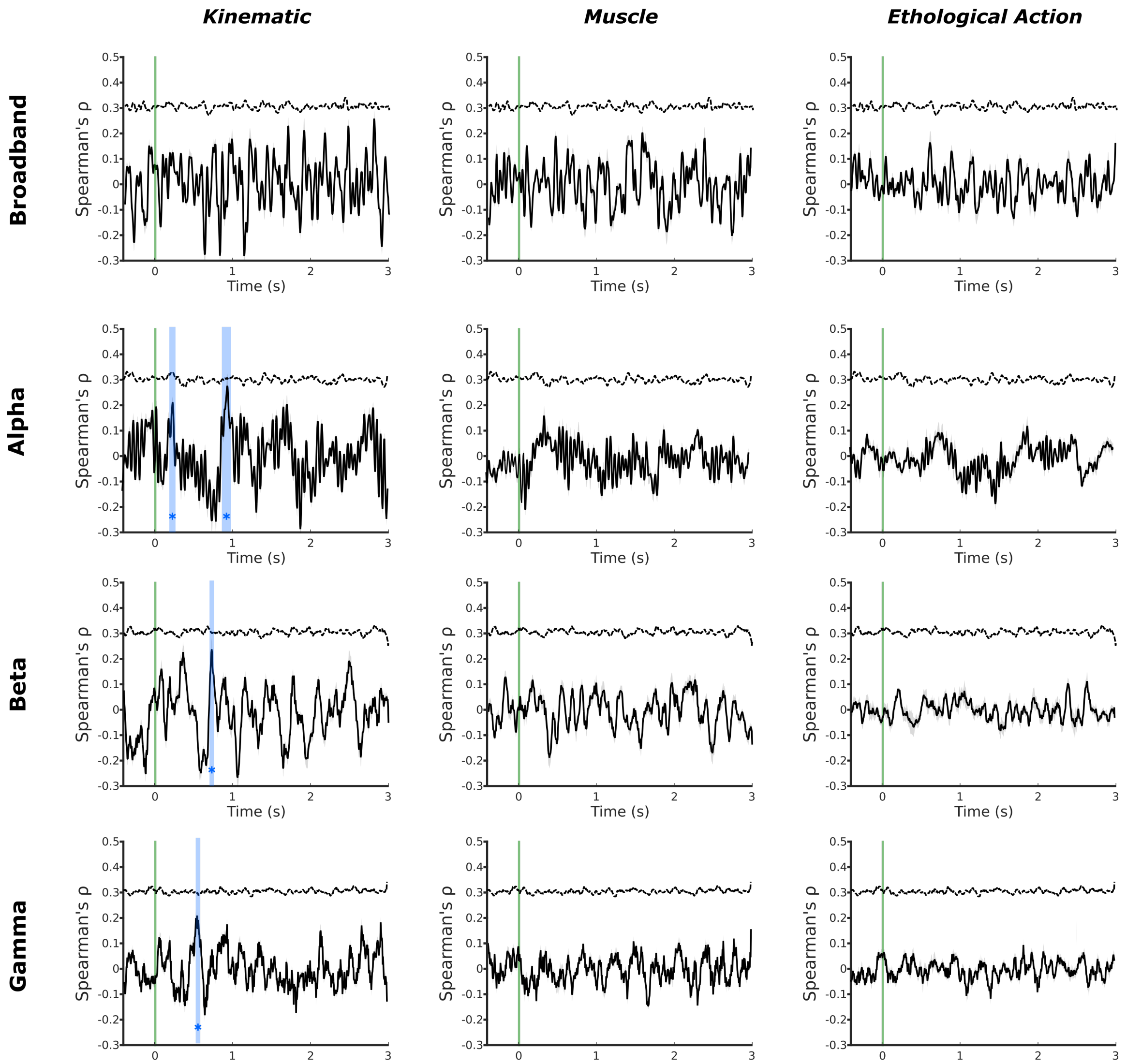

Figure S4: MEG searchlight analysis during action observation Evidence of a significant peak in the correspondence between the kinematic model and the alpha frequency MEG signal (220-255ms and 890-955ms), the beta band MEG signal (705-735ms) and the gamma band MEG signal (545-560ms) in the period of action observation. No equivalent concurrence with the MEG signal was observed for the muscle of ethological action model in primary motor cortex during action observation. The green line indicates the onset of the stimulus video; the blue regions indicate significant peaks in representational similarity between MEG data and the motor model; the dashed line indicates noise ceiling. Comparison with MEG temporal searchlight results presented in Figures 1 and 3.

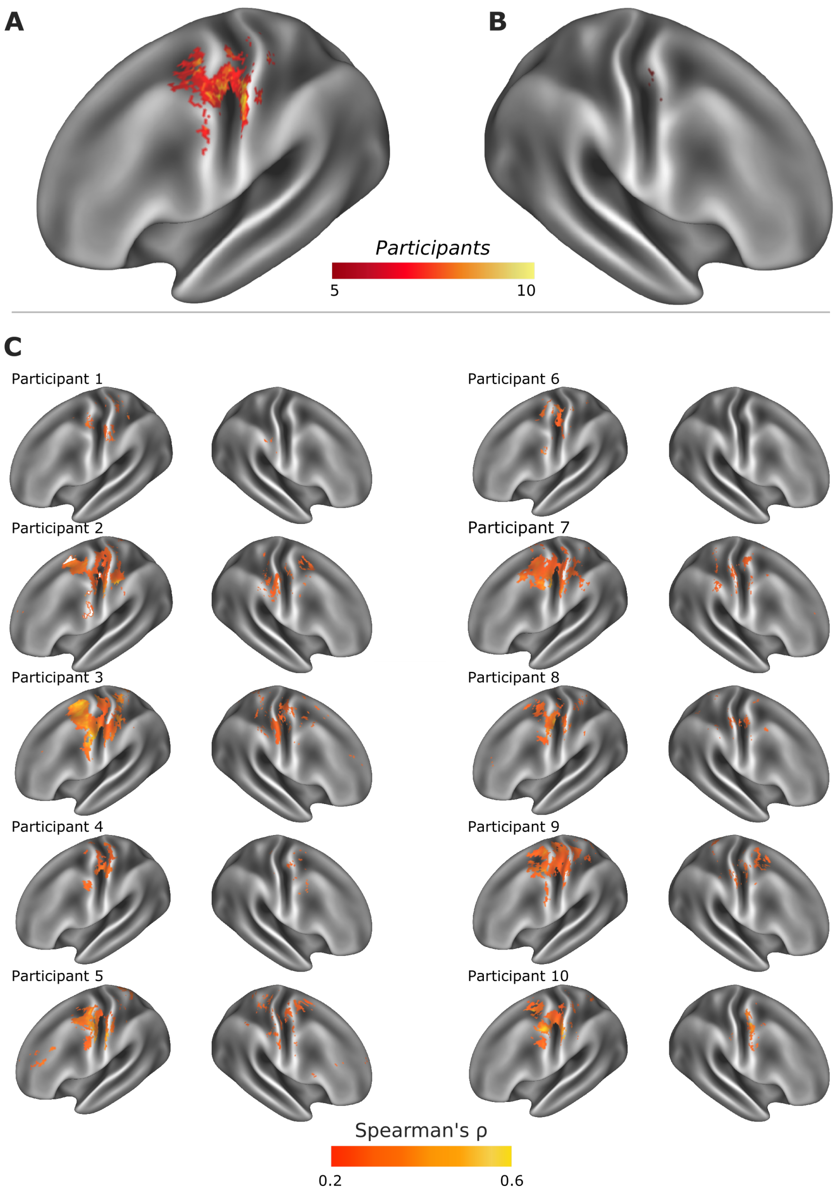

Figure S5: Individual participant cortical searchlight results using a muscle model of movement encoding. Cortical heatmaps of the left (A) and right (B) hemisphere, show consistent ncoding of an action model based on EMG recordings in Brodmann areas 4 and 3b. Heatmaps were constructed from individually thresholded cortical searchlights for each participant using a single average muscle model (C) (Omnibus threshold, $\alpha = 0.01$, maximum accuracy distribution calculated from peak correlation value across 10,000 searchlight permutations with label-switching.)

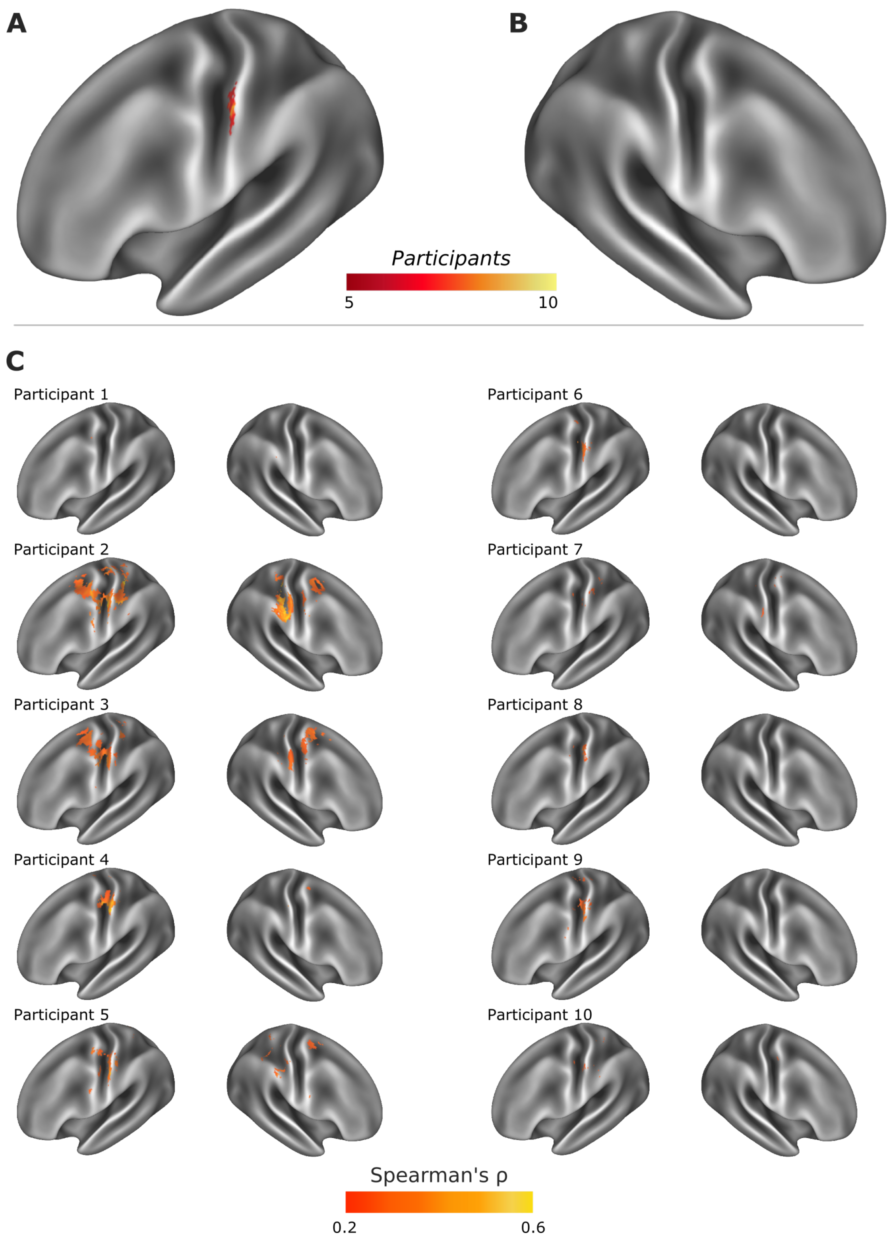

Figure S6: Individual participant cortical searchlight results using an ethological action model of movement encoding. Cortical heatmaps of the left (A) and right (B) hemisphere, show limited but consistent encoding of an action model in the left post-central gyrus, contralateral to movement. Heatmaps were constructed from individually thresholded cortical searchlights for each participant using a single categorical action model (C) (Omnibus threshold, $\alpha = 0.01$, maximum accuracy distribution calculated from peak correlation value across 10,000 searchlight permutations with label-switching.)

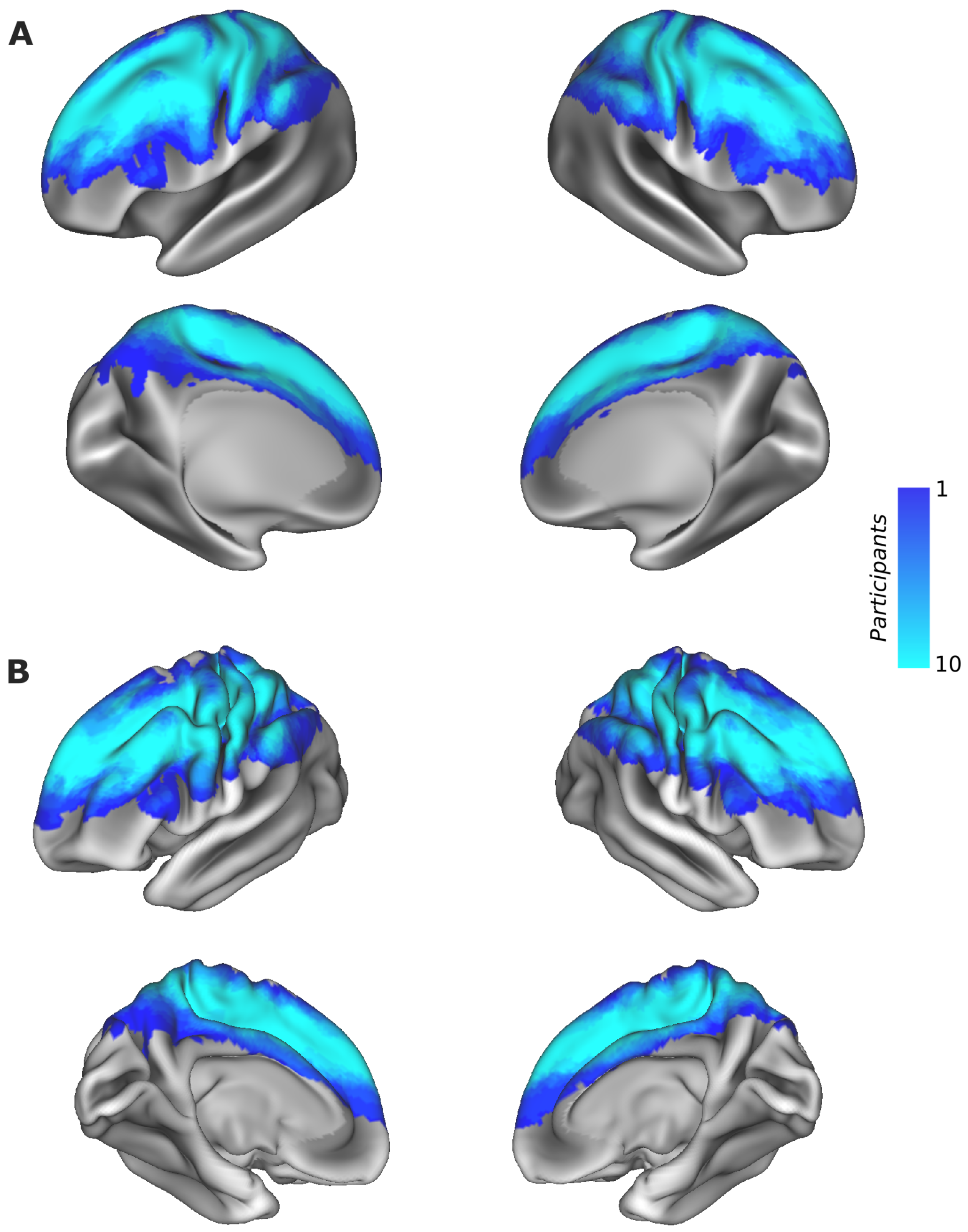

Figure S7: Heatmap surface visualisation of fMRI data coverage across participants on inflated (A) and midthickness (B) surfaces.

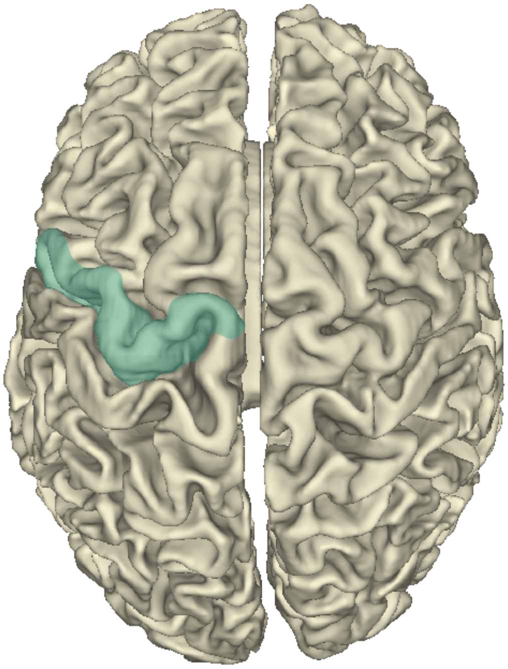

Figure S8: Surface visualisation of the left hemisphere motor region of the AAL atlas used in MEG temporal searchlight analysis: precentral L.

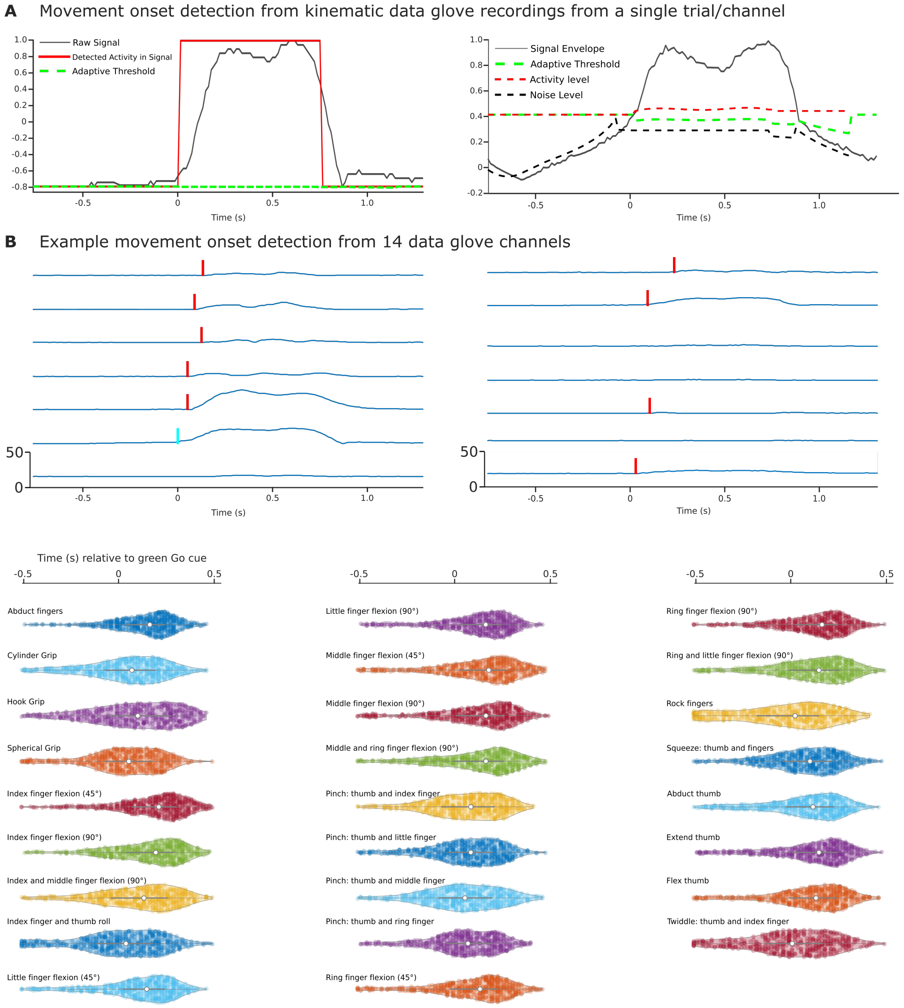

Figure S9: Movement onset time calculations from data glove recordings used to epoch MEG recordings. A. Example of data glove signal onset detection using adaptive threshold for one of the 14 channels on a single trial. The unsmoothed envelope of signal (Hilbert transform) was used for automatic onset detection. B. Illustration of movement onset detection in the same trial across all 14 channels; movement onset was detected in a subset of channels (red); the earliest of these (cyan) was used to conservatively estimate the onset of movement, and was therefore defined as timepoint 0 s. C. Distribution of data glove onset times for all trials across all participants in the 26 different movement categories; time point 0 s indicates the onset of the green visual Go cue: these glove onset times were used to epoch MEG data to account for inter-trial variability in the representational similarity analysis

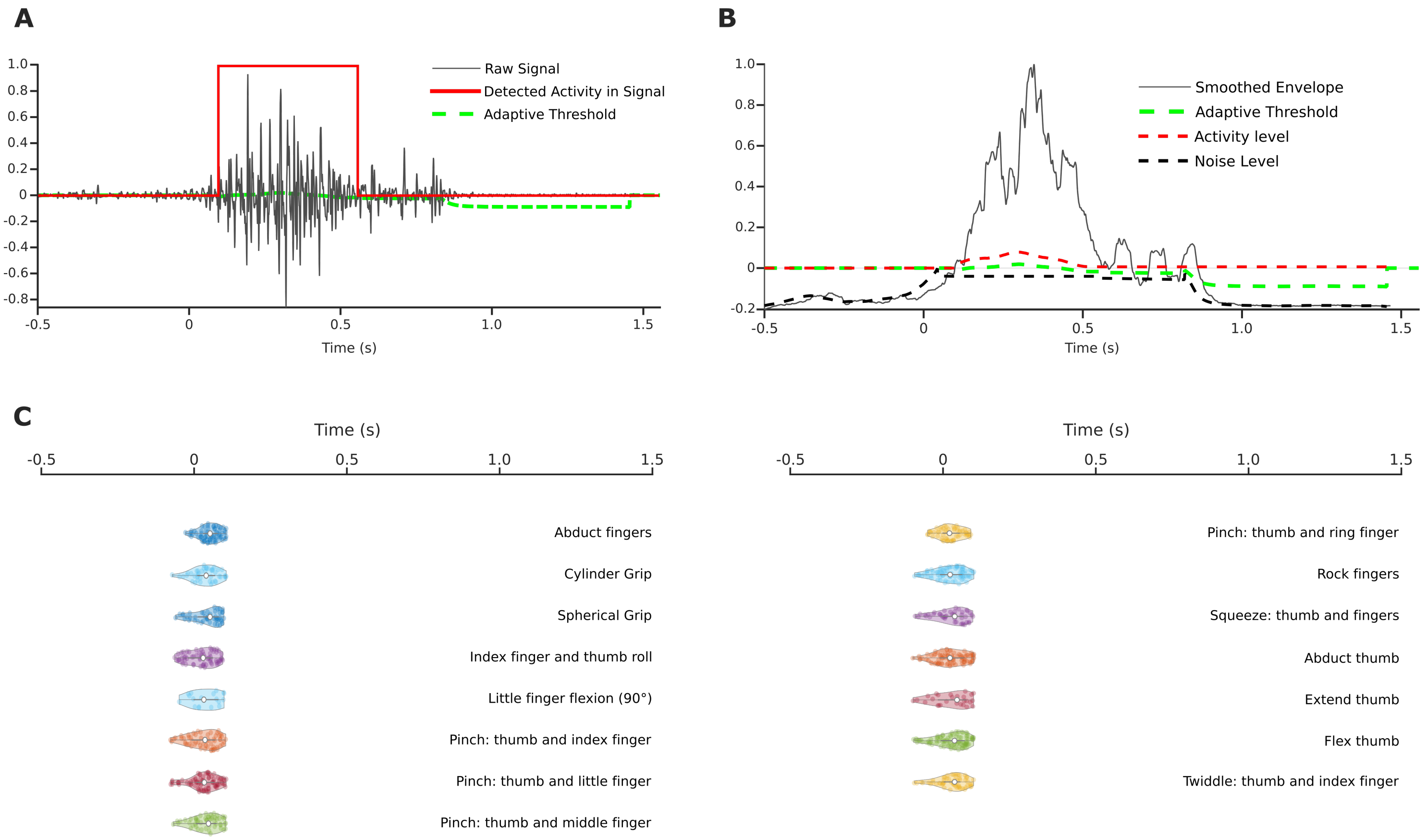

Figure S10: EMG muscle activity onset time. A. Example of EMG onset detection using adaptive threshold. B. Smoothed envelope of signal (Hilbert transform; 5ms smoothing window) used for automatic onset detection. C. Violin plots visualising the distribution of EMG onset times for trials from which onset could be detected across all participants. Bars represent interquartile range, white dots represent median values which are plotted in Figure 1. EMG data were used to exclude trials where muscle onset time differed from data glove defined movement onset time by $> \pm$ 100 ms.

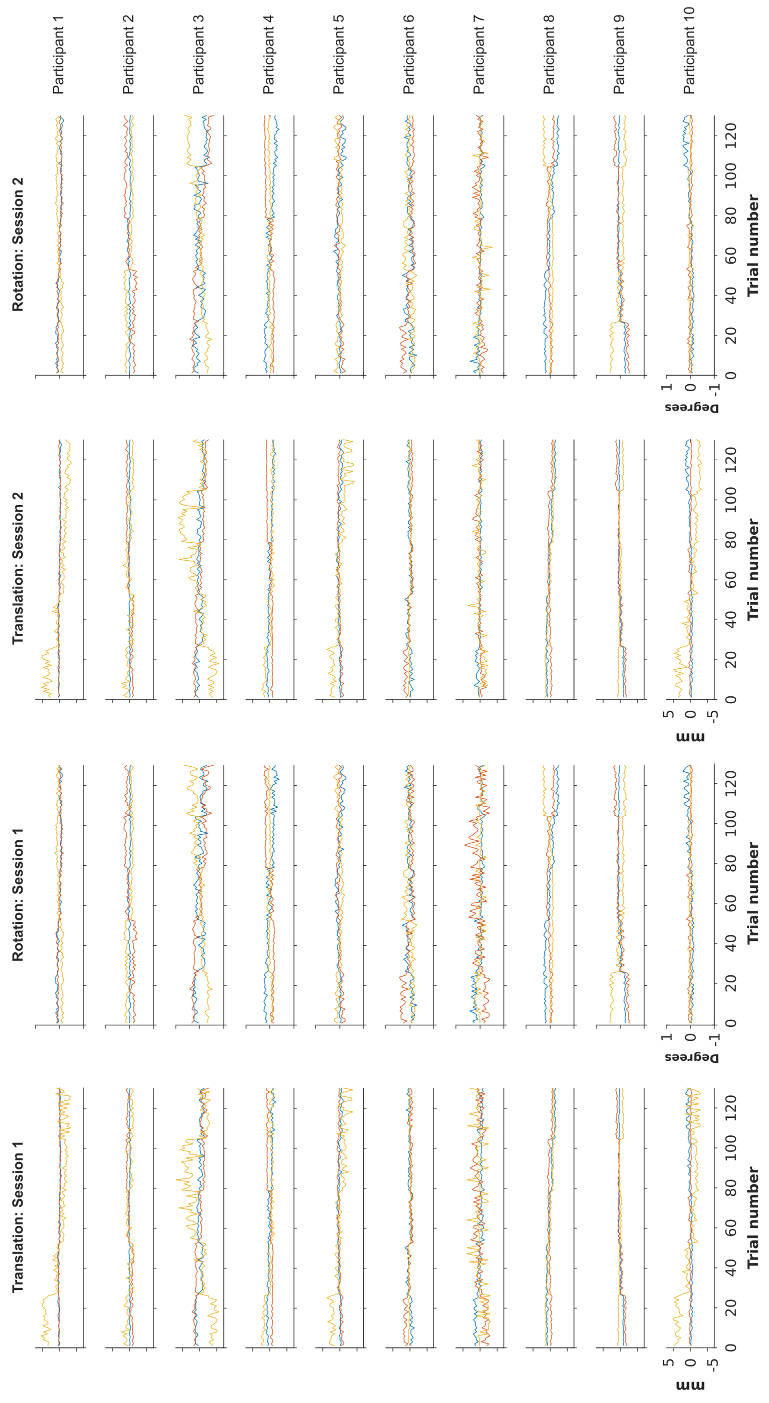

Figure S11: MEG motion timeseries across participants and sessions. Blue: X-axis, orange: Y-axis, yellow: Z-axis

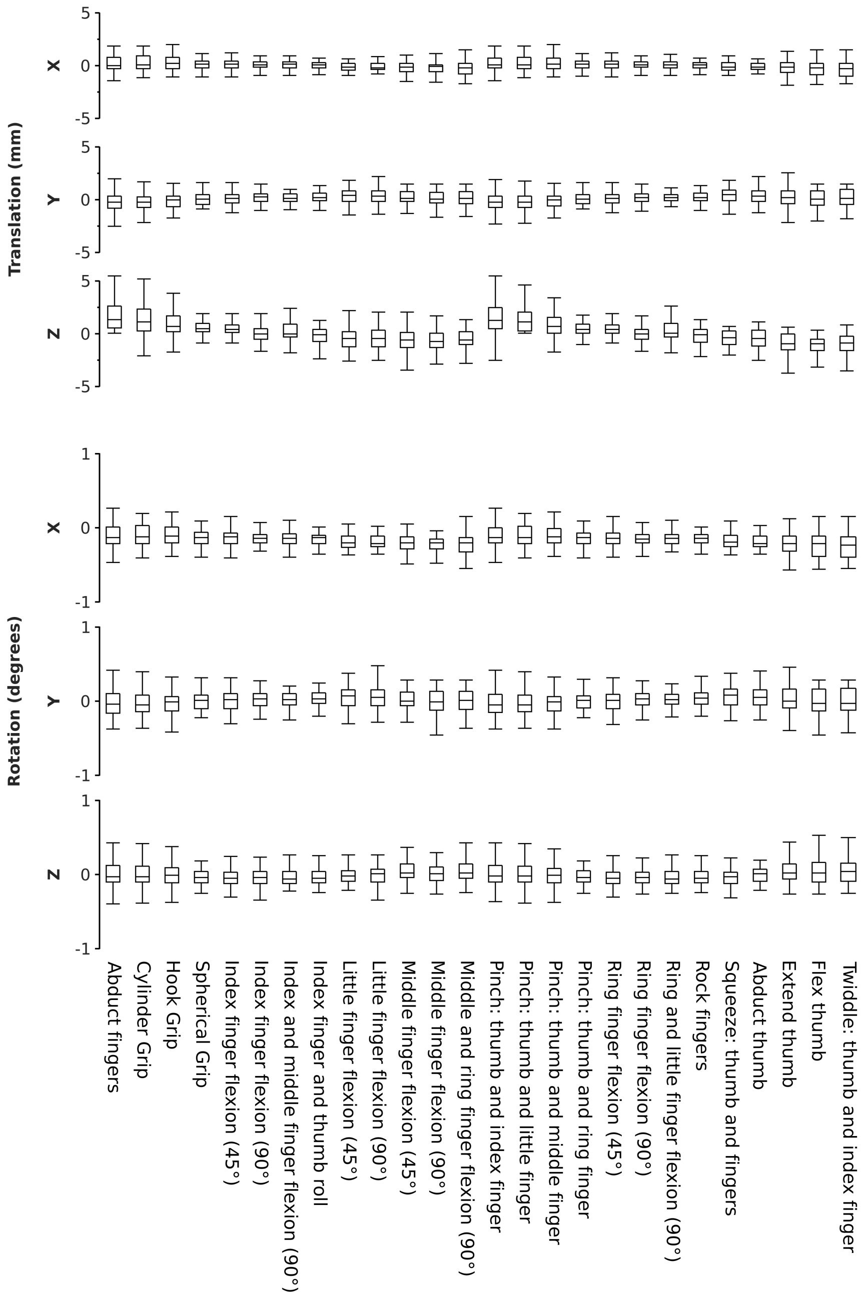

Figure S12: MEG motion comparison across movement conditions.

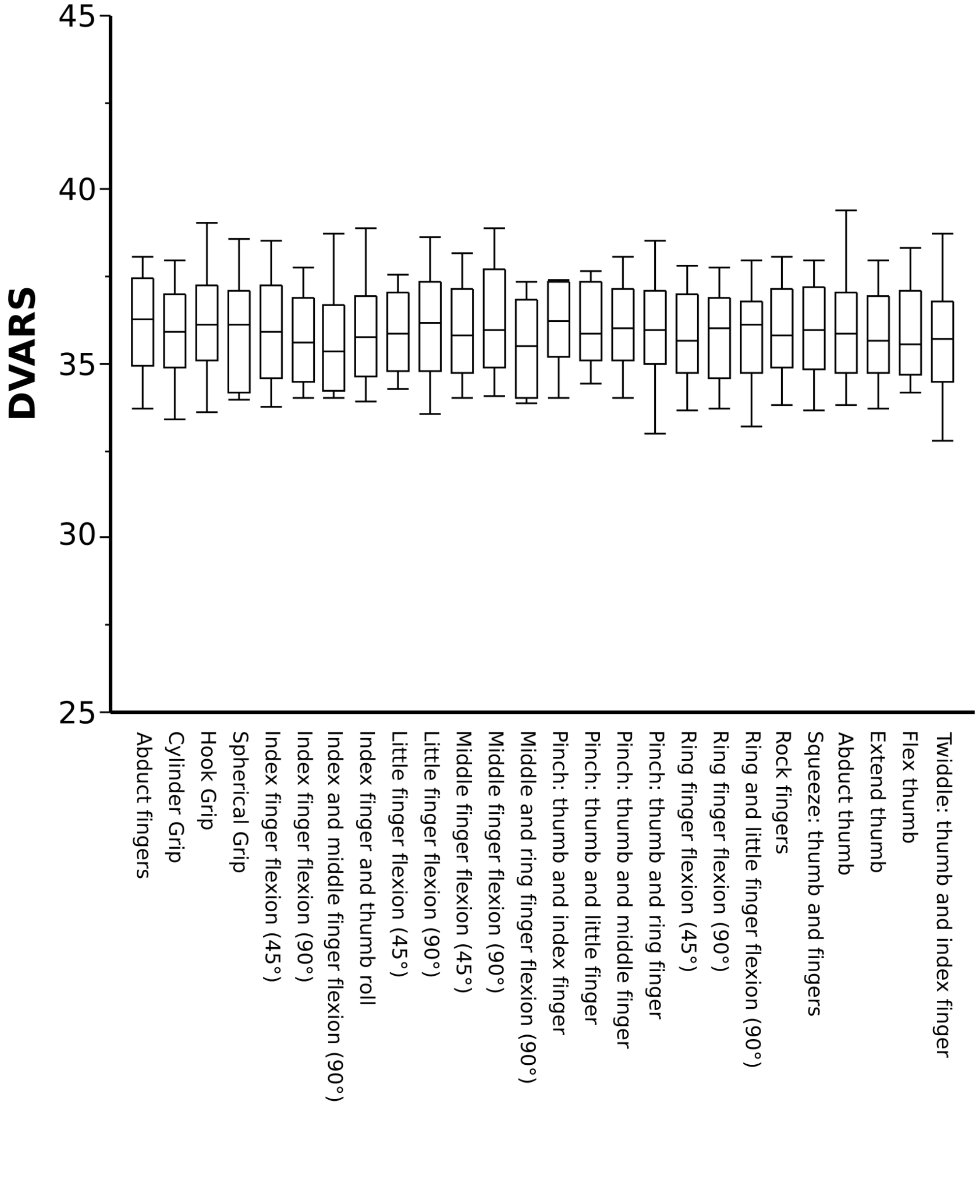

Figure S13: fMRI motion comparison across movement conditions using DVARS presented in arbitrary units (Power et al., 2012, 2014; Afyouni & Nichols, 2018).

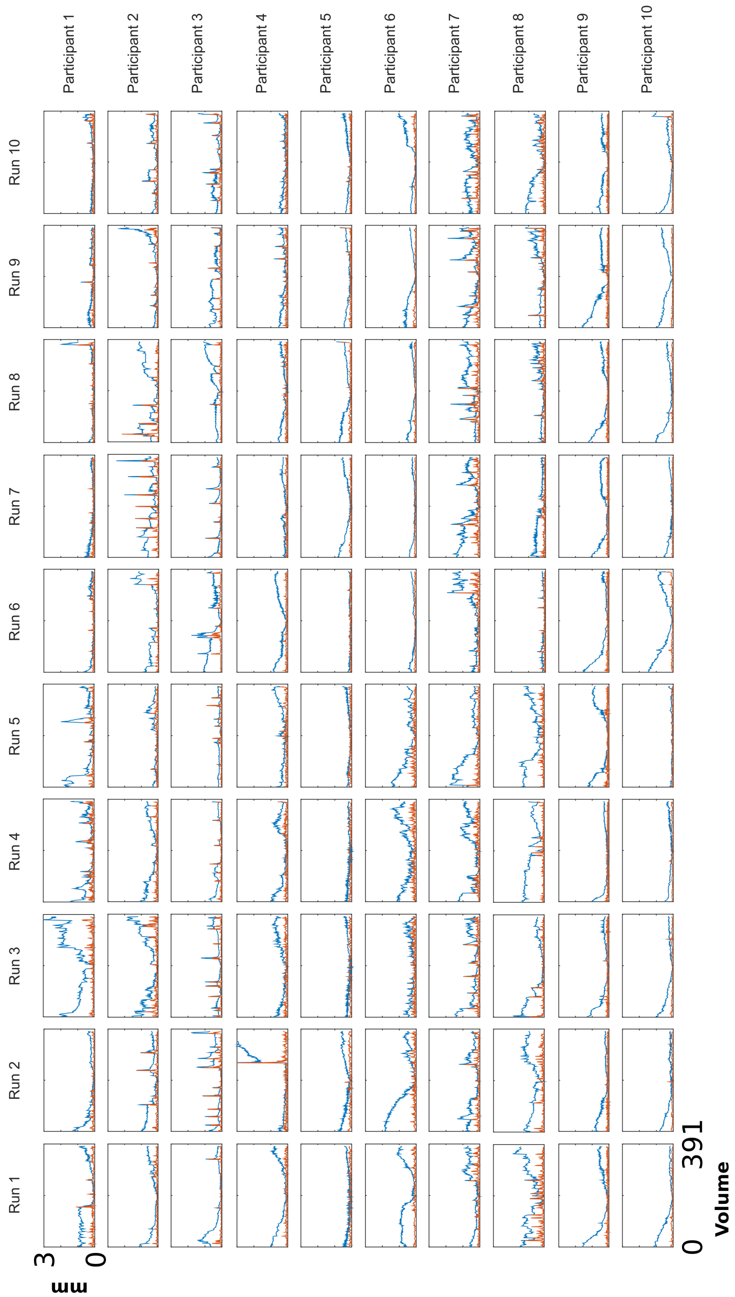

Figure S14: fMRI motion displacement plots. Plots of absolute (blue) and relative (orange) motion calculated using FSL MCFLIRT for each participant and each fMRI task run; motion correction was undertaken prior to ICA denoising.

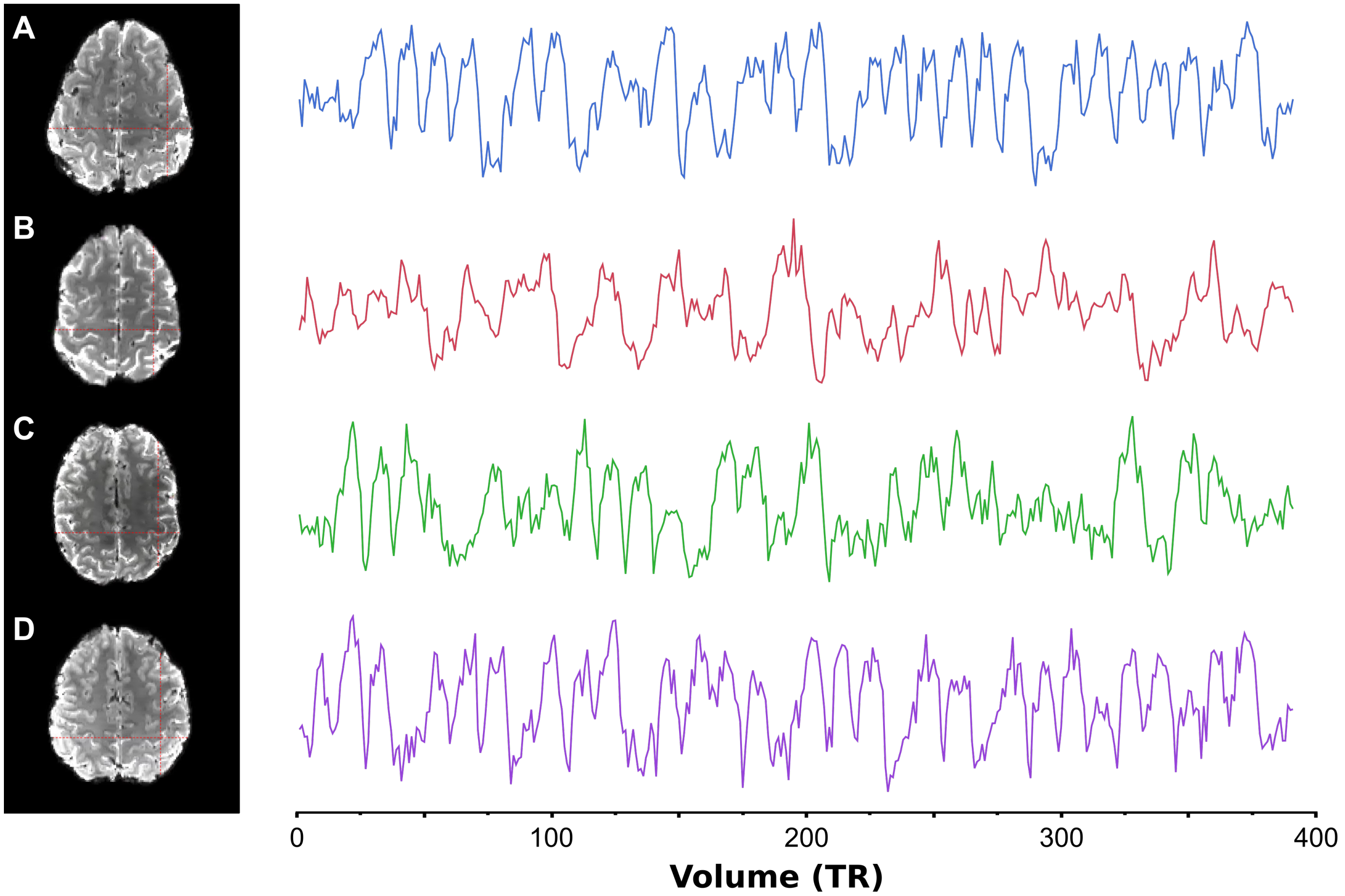

Figure S15: Example fMRI axial slices and single voxel timeseries from 4 participants. fMRI timeseries data extracted from a single voxel (red crosshairs) for four participants. Data presented were subject to high-pass filter (100 seconds). fMRI data were not subject to spatial or temporal smoothing.

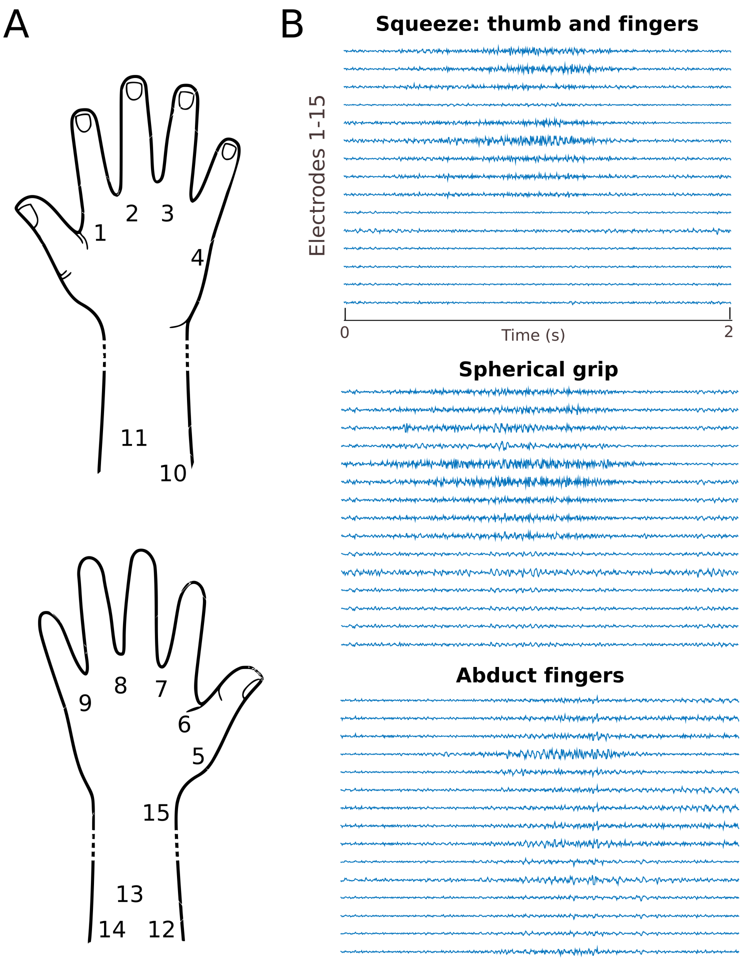

Figure S16: EMG recordings from an independent cohort used to generate a muscle model of hand movement. A) Schematic demonstrating electrode placement for EMG recordings on both the palmar and dorsal surface of the hand and forearm covering these muscles: 1: first dorsal interosseus (FDI), 2-3: dorsal interosseus muscles, 4: abductor digiti minimi, 5: abductor pollicis brevis (APB), 6: adductor pollicis, 7-9: lumbrical muscles, 10: flexor carpi ulnaris, 11: flexor carpi radialis, 12-14: flexor digitorum superficialis and flexor digitorum profundus, 15: flexor pollicis longus. Dotted lines indicate region of the forearm compressed for visualisation purposes. This montage replicated that used in previous studies of kinematic and muscle information encoding in M1 (Ejaz et al., 2015; Leo et al., 2016). B) Example EMG trace from one participant for three different movements (squeeze, spherical grip and abduct fingers) showing electrodes 1-15 across the 2s movement time window.

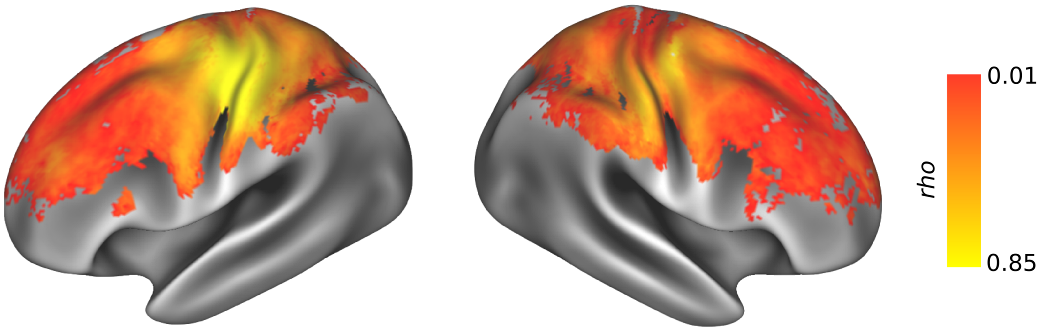

Figure S17: Noise ceiling calculation for spatial searchlight using fMRI data. To assess the spatial consistency in RDMs calculated from fMRI data at each vertex, each participant's RDM was correlated with the average cross-subject RDM; the correlations were then averaged to obtain a vertex-wise upper bound of the noise ceiling.

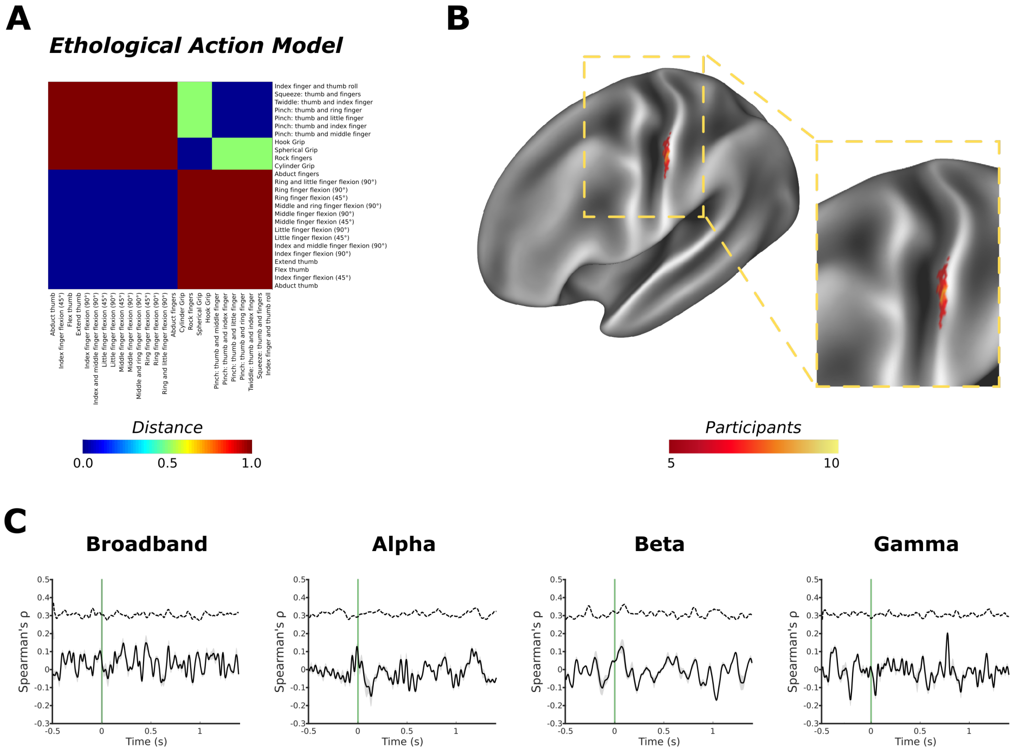

Figure S18: Ethological action model reveals limited evidence for spatial encoding in primary somatosensory cortex. (A) A theoretical model of cortical movement encoding on the basis of ethological action type was constructed on the basis of compelling evidence for such functional organisation in M1 from the primate literature (Graziano, 2016). (B) The spatial searchlight conducted using fMRI data revealed consistent cortical encoding of information in the post-central gyrus (Brodmann area 3b). (C) No evidence for the temporal encoding of this model was observed from MEG analysis.

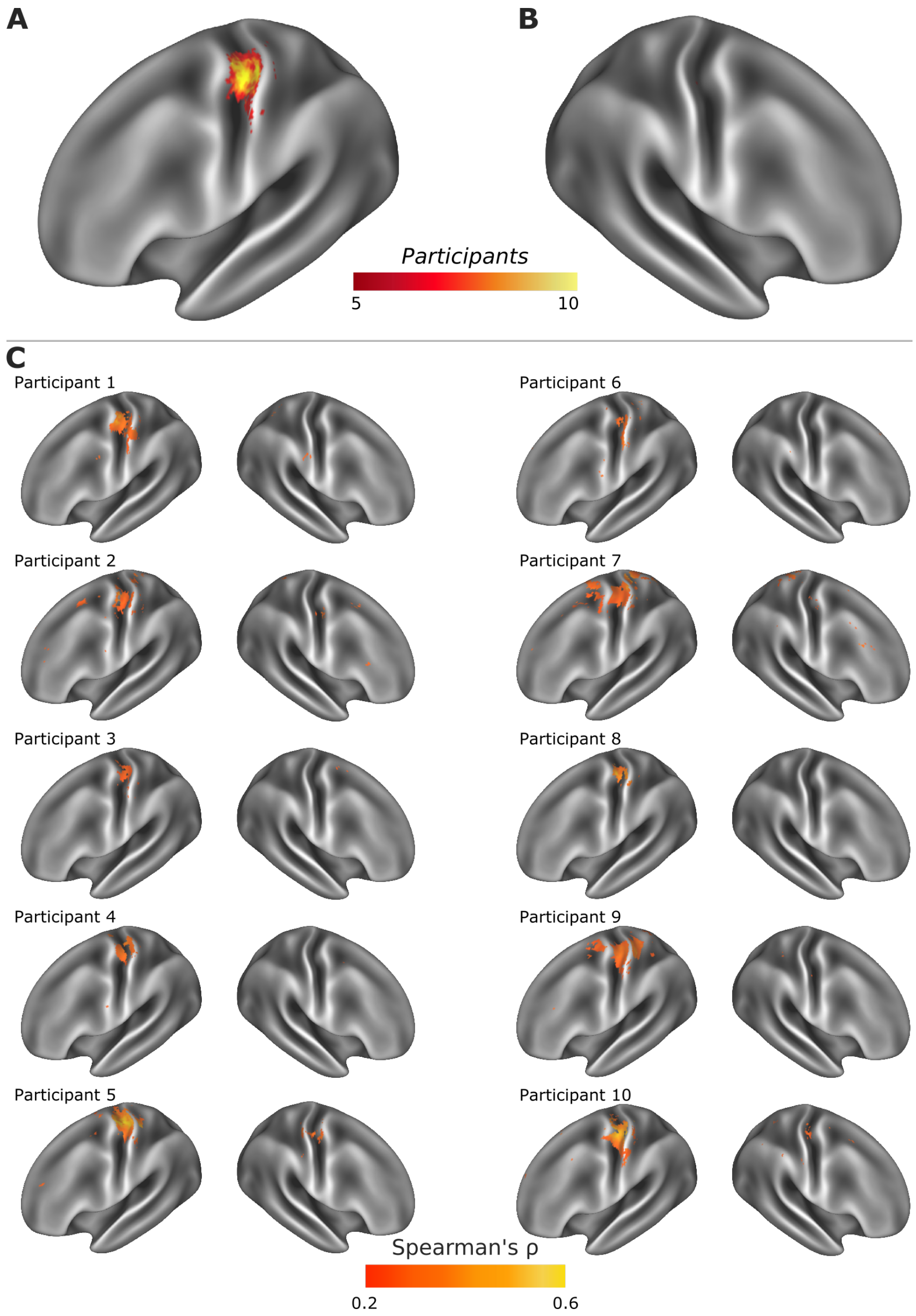

Figure S19: fMRI searchlight analysis conducted using the kinematic model constructed from data glove recordings made during the behavioural testing session. Evidence of the encoding of kinematic data in contralateral primary motor cortex persists using independent data glove recordings while participants were sitting upright in a more naturalistic position. Comparison with data presented in Figures 1 and 2.

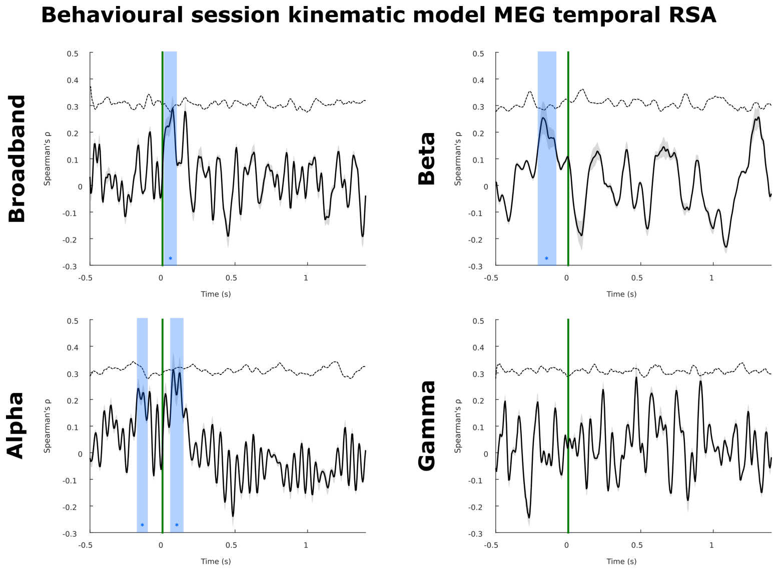

Figure S20: Temporal searchlight analysis investigating information encoding produces consistent results when using data glove recordings acquired during the behavioural testing session, when compared against the equivalent model derived from kinematic recordings made during the MEG sessions (Figure 3). Green line - movement onset defined by the data glove; blue regions - significant peaks in representational similarity between MEG data and the motor model; dashed line - correlation noise ceiling.

.

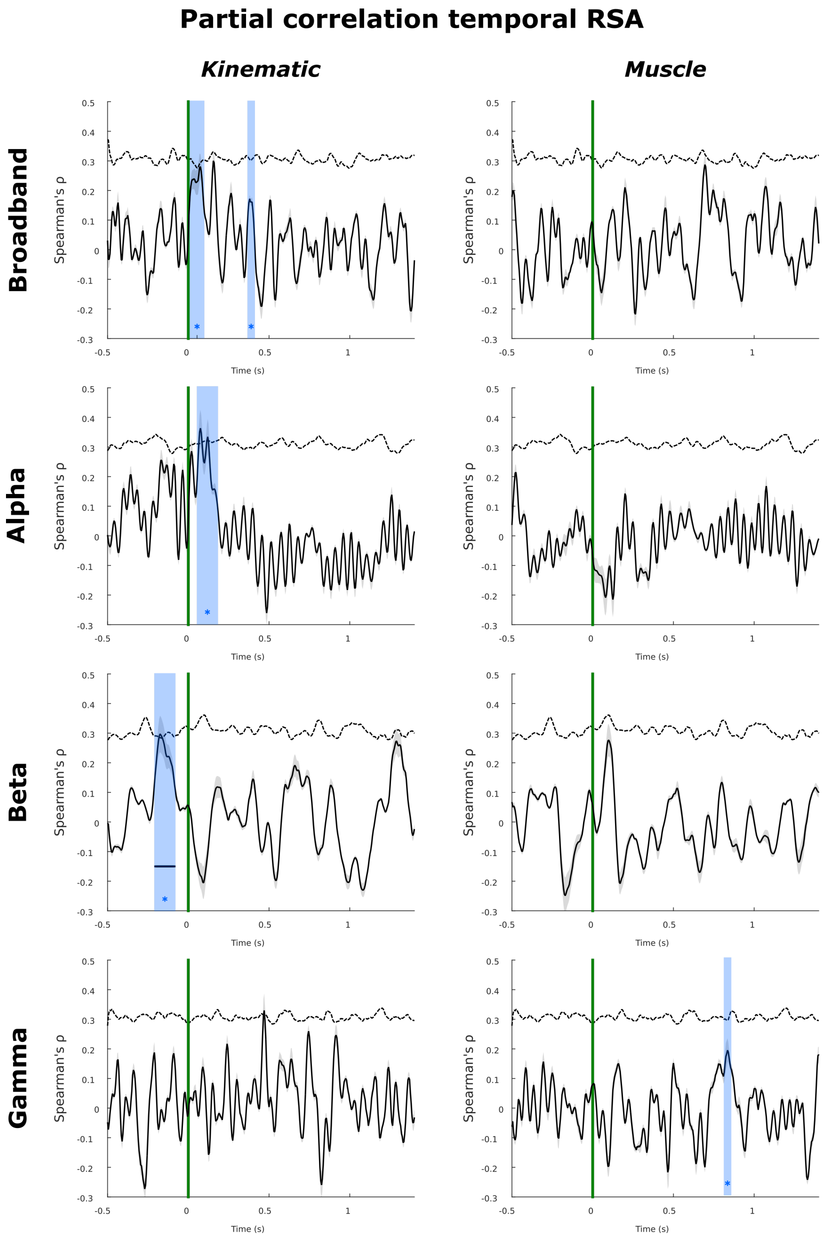

Figure S21: Temporal searchlight analysis using partial correlations contrasting kinematic and muscle models reveal results comparable to original Spearman's ρ analysis presented in Figure 3. Green line - movement onset defined by the data glove; blue regions - significant peaks in representational similarity between MEG data and the model (1000 shuffled permutations of candidate model RDMs; cluster-forming threshold: $p< 0.01$; maximal cluster distribution ($\alpha = 0.001$)) ; dashed line - correlation noise ceiling.
